# Genome-wide identification of the superoxide dismutase gene family in *Lycium barbarum* and their expression profiles under abiotic stress and phytohormone treatment

**DOI:** 10.3389/fpls.2026.1897912

**Published:** 2026-08-27

**Authors:** Xiaoyu Cao, Xiaorong Bai, Yingwu Li, Xuedong Hui, Jinfeng Niu, Xiaojie Liang, Yue Yin, Wei An

**Affiliations:** 1 National Wolfberry Engineering Research Center, NingXia Academy of Agriculture and Forestry Sciences, Yinchuan, China; 2 General Station of State-owned Forest Farms and Tree Seedlings, Ningxia Forestry and Grassland Administration, Yinchuan, China

**Keywords:** abiotic stress, expression pattern, Lycium barbarum, phylogenetic analysis, phytohormones, superoxide dismutase

## Abstract

**Background:**

Superoxide dismutases (SODs) are crucial metalloenzymes that constitute the first line of defense against reactive oxygen species in plants under abiotic stress. Wolfberry (*Lycium barbarum*) is an economically important medicinal plant with notable stress tolerance, however, a comprehensive genome-wide analysis of its SOD gene family has not yet been performed.

**Results:**

We identified ten wolfberry *SOD* genes (*LbaSODs*) and classified them into three subfamilies: iron-SODs (Fe-SODs), manganese-SODs (Mn-SODs), and copper/zinc-SODs (Cu/Zn-SODs). Members within each subfamily shared conserved gene structures and motifs. Segmental duplication was the primary driver of *LbaSOD* expansion, with three paralogous pairs identified. Analysis of *cis*-regulatory elements in the promoter region revealed a predominance of stress- and hormone-responsive *cis*-elements, particularly ABA-responsive elements (ABREs) (22 copies) and LTR (17 copies) motifs. Tissue-specific expression profiling revealed that *LbaSOD2* and *LbaSOD5* expression peaked during early fruit development, whereas *LbaSOD6*, *LbaSOD9*, and *LbaSOD10* were progressively upregulated through fruit maturation. Under abiotic conditions, Fe-SOD members were markedly suppressed during prolonged drought, whereas *LbaSOD9* and *LbaSOD10* were rapidly induced in response to salt stress. Among the phytohormone treatments, methyl jasmonate (MeJA) elicited the most pronounced response, with *LbaSOD5* expression increasing by approximately 60-fold after 24 hours. Notably, abscisic acid (ABA) triggered an exceptionally strong transcriptional induction of *LbaSOD5* (2.5 × 10^5^-fold), *LbaSOD10* (6 × 10^5^-fold), and *LbaSOD6* (70-fold). In addition, *LbaSOD3* and *LbaSOD7* transcripts were undetectable in any of the tested conditions.

**Conclusions:**

This study provides the first comprehensive characterization of the *LbaSOD* gene family and elucidates its hormone- and stress-responsive regulatory landscape, providing a valuable foundation for future functional investigations of *LbaSOD* genes in abiotic stress adaptation. The extraordinarily strong ABA-mediated induction of specific *LbaSOD* members, together with their tissue- and stress-specific expression patterns, highlights their potential as targets for genetic improvement of stress tolerance in wolfberry.

## 1 Introduction

Wolfberry (*Lycium barbarum* L.), a perennial shrub of the *Solanaceae* family, produces nutrient-dense fruits valued for their high concentration of polysaccharides, carotenoids, and antioxidants (Amagase and Farnsworth, 2011; Inbaraj et al., 2008; Potterat, 2010). These fruits have been used for centuries in traditional medicine to enhance immunity, improve vision, and protect against oxidative damage (Bucheli et al., 2011; Yao et al., 2018). However, wolfberry cultivation is frequently constrained by abiotic stresses, including drought, high salinity, and extreme temperatures (Zhao et al., 2018; Liang et al., 2025), which trigger the overproduction of reactive oxygen species (ROS) such as superoxide anions (O_2_^–^), hydrogen peroxide (H_2_O_2_), and hydroxyl radicals (OH), causing oxidative damage to lipids, proteins, and nucleic acids (Apel and Hirt, 2004; Mittler, 2017).

Superoxide dismutases (SODs) constitute the first line of enzymatic antioxidant defense, catalyzing the dismutation of O_2_^–^ into O_2_ and H_2_O_2_ (Gill and Tuteja, 2010; Mittler, 2002). Plant SODs are classified into three subfamilies based on their metal cofactors: copper/zinc-SODs (Cu/Zn-SODs), iron-SODs (Fe-SODs), and manganese-SODs (Mn-SODs), which are compartmentalized in the cytosol, chloroplasts, mitochondria, and apoplast, thereby enabling spatially organized ROS scavenging (Mishra and Sharma, 2019; Verma et al., 2019). Genome-wide analyses of SOD gene families have been conducted in model and crop species, including *Arabidopsis thaliana* (Kliebenstein et al., 1998), rice (Yadav et al., 2019), tomato (Feng et al., 2016), wheat (Tyagi et al., 2017), and maize (Liu et al., 2021). Comprehensive characterization of the SOD gene family has been reported in tomato (Feng et al., 2016), potato (Rudić et al., 2022), and tobacco (Huo et al., 2022), revealing conserved subfamily compositions and species-specific regulatory patterns. Recently, genome-wide analyses of SOD genes have been extended to a wide range of plant lineages, including peanut (Yu et al., 2023), pakchoi (Zhou et al., 2024), ginkgo (Song et al., 2024), and medicinal species, such as balloon flower (Hyun, 2025) and the drought-tolerant argan tree (Chahidi et al., 2025), highlighting the broadening scope of SOD research across both ecological and economic contexts.

The expression of plant SOD genes is tightly regulated by phytohormone signaling networks, particularly abscisic acid (ABA), MeJA, salicylic acid (SA), and ethylene (ET), which integrate ROS homeostasis with stress responses (Mittler et al., 2011; Xia et al., 2015). ABA-induced upregulation of SODs has been documented in rice and *Arabidopsis* under drought and salinity (Tsugane et al., 1999; Wang et al., 2005), whereas MeJA and SA modulate SOD expression during pathogen defense and systemic acquired resistance (Dat et al., 1998; Orozco-Cárdenas et al., 2001). However, the extent to which multiple phytohormones coordinately regulate the entire SOD gene family within a single species and whether these regulatory mechanisms differ among plant lineages remains largely unclear. Most previous studies have focused on individual phytohormones or a limited number of SOD genes, leaving the family-wide regulatory network largely uncharacterized in the literature.

Despite the well-documented antioxidant properties of wolfberry fruits (Li et al., 2007; Wu et al., 2010), a comprehensive genome-wide characterization of the SOD gene family has not yet been performed. Notably, although SOD gene families have been characterized in several annual *Solanaceae* crops, including tomato, potato, and tobacco, wolfberry is the first perennial medicinal member of the *Solanaceae* species to be examined, offering a unique evolutionary and ecological context. Unlike annual crops, the perennial growth habit of wolfberry, together with its adaptation to the arid and semi-arid environments of northwestern China, subjects the species to prolonged and recurrent oxidative stress, potentially driving the evolution of distinctive regulatory mechanisms within the antioxidant gene network. Furthermore, existing studies on the SOD family have typically examined gene expression under only one or two phytohormone treatments. However, a systematic comparison of the entire SOD gene family across multiple phytohormone classes within a single species remains rare, limiting our understanding of how hormone signaling networks coordinately regulate this critical gene family. The recent release of a chromosome-level wolfberry genome assembly (Cao et al., 2021) now enables a comprehensive analysis.

In this study, we aimed to (1) identify and classify all *LbaSOD* genes in the wolfberry genome and characterize their phylogenetic relationships, gene structures, conserved motifs, and chromosomal distribution; (2) analyze gene duplication events and interspecific synteny to infer the evolutionary drivers of family expansion; (3) catalog *cis*-regulatory elements in *LbaSOD* promoters; (4) profile *LbaSOD* expression across tissues during fruit developmental stages; and (5) systematically compare their transcriptional responses to three abiotic stresses (cold, salt, and drought) and five phytohormones (ABA, indole-3-acetic acid [IAA], gibberellic acid [GA], SA, and MeJA), with particular attention to hormone-specific and subfamily-specific regulatory patterns.

## 2 Materials and methods

### 2.1 Plant materials

Root, stem, leaf, flower, and fruit samples of wolfberry were collected from the experimental fields of the National Wolfberry Engineering Research Centre, Yinchuan, China. Fruit developmental stages were defined based on days after flowering (DAF) and classified as 12 DAF (initial ripening), 19 DAF (early maturation), 25 DAF (mid-maturation), 30 DAF (full maturation) and 37 DAF (post-maturation). All samples were promptly frozen in liquid nitrogen and stored at −80 °C until further processing.

### 2.2 Identification and characterization of *LbaSOD*s in the wolfberry genome

To comprehensively identify *LbaSOD*s in wolfberry, we implemented a dual-strategy approach. First, BLASTP analysis was performed against the wolfberry proteome using *A. thaliana* SOD protein sequences from the TAIR database (https://www.arabidopsis.org/, accessed 15 January 2024) as queries, with an E-value cutoff of 1 × 10^–5^. Second, Hidden Markov Model searches were performed using HMMER 3.0 with default parameters (E-value < 0.001) against the wolfberry proteome, utilizing Pfam domains PF00080, PF00081, and PF02777 (https://www.ebi.ac.uk/interpro/, accessed 15 January 2024). Candidate genes were subsequently validated for characteristic SOD domains using SMART (https://smart.embl.de/, accessed 15 January 2024), Pfam, and the NCBI Conserved Domain Database (https://www.ncbi.nlm.nih.gov/cdd, accessed 15 January 2024). Proteins containing conserved SOD domains were designated as LbaSODs. To eliminate redundancy, candidate genes were clustered using CD-HIT with a sequence identity threshold of 95%, and only non-redundant loci were retained. Key biochemical properties, including amino acid length, molecular weight, isoelectric point, and GRAVY, were calculated for each validated LbaSOD using the ProtParam tool available through ExPASy (https://web.expasy.org/protparam/, accessed 15 January 2024).

### 2.3 Phylogenetic tree construction and structural analysis of *LbaSODs*

**Figure 1:**
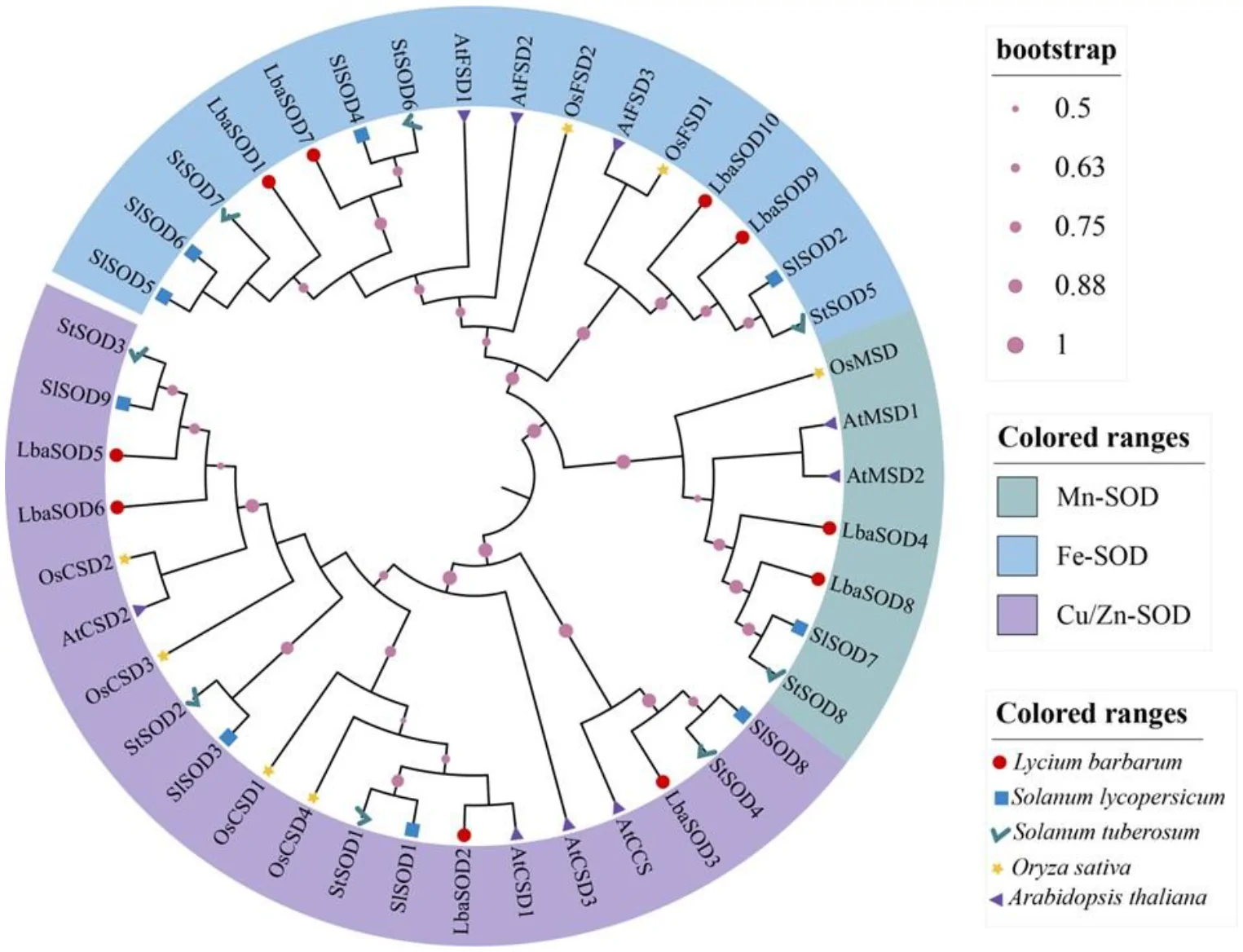
Phylogenetic tree constructed using SODs from *S. lycopersicum* (*SlSOD*), *S. tuberosum* (*StSOD*), *A. thaliana* (*AtSOD*), *O. sativa* (*OsSOD*) and *L. barbarum* (*LbaSOD*), indicating the Mn-SOD (green), Fe-SOD (light blue), and Cu/Zn-SOD (purple) subfamily groups. The tree was constructed using MEGA 6.06 with the Maximum Likelihood method and 1000 bootstraps.

**Figure 2:**
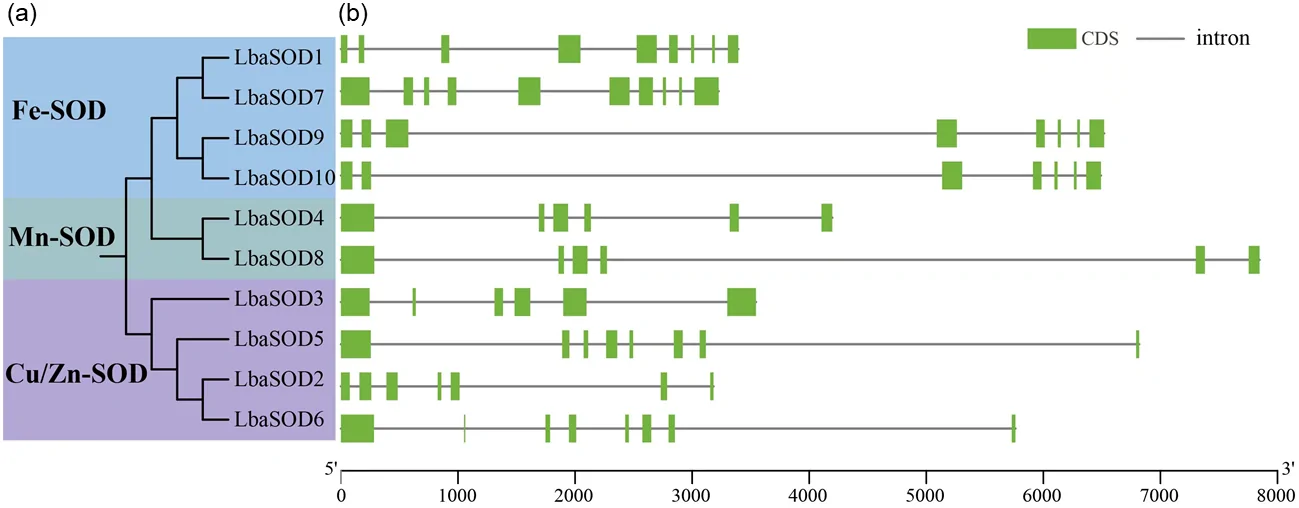
Phylogenetic tree of the *LbaSOD* family constructed using the Maximum Likelihood method and 1000 bootstraps **(a)** and gene structure of *LbaSOD*s **(b)**. The green rectangle indicates an exon, and the black line indicates an intron. The scale bar indicates 1 kb.

**Figure 3:**
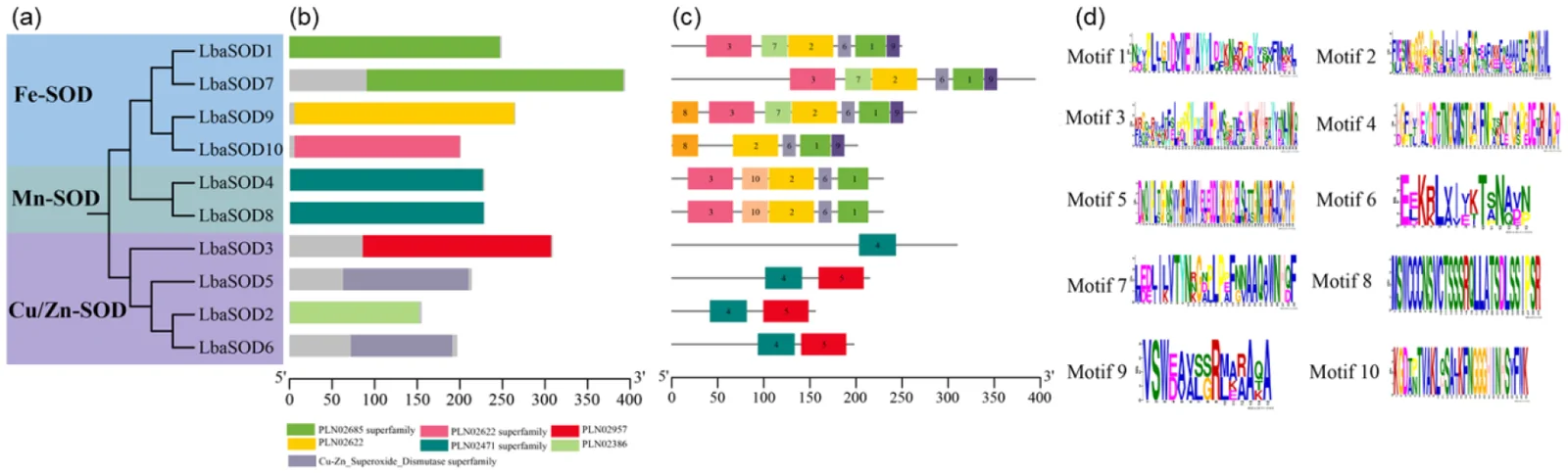
Phylogenetic classification, conserved domain distribution, conserved motif arrangement, and motif sequence logos of LbaSOD proteins. **(A)** Phylogenetic tree of LbaSODs. **(B)** Visualization of the conserved Pfam superfamily domain distribution of each LbaSOD protein. Colored rectangular blocks represent different conserved superfamily domains, with the corresponding legends displayed at the bottom. **(C)** Distribution of conserved motifs across the 10 LbaSODs. In the present study, ten motifs were predicted using MEME. The scale bar represents 50 nucleotides. **(D)** Detailed amino acid sequences of the 10 conserved motifs, with colored and sized letters used to visually distinguish between different residues. The sequences correspond to the MEME-predicted motifs shown in **Figure 3B**.

Sequences of AtSODs were obtained from the TAIR database (https://www.arabidopsis.org/, accessed 25 January 2024). Genes for *Solanum lycopersicum* and *S. tuberosum* were retrieved from the Solanaceae Genomics Network platform (https://solgenomics.net/, accessed 25 January 2024), and orthologous sequences for *Oryza sativa* were compiled from previous studies (Gill and Tuteja, 2010). The SOD sequences were globally aligned using MUSCLE v5 (Miller et al., 2009), and evolutionary relationships were inferred using MEGA 6.06 (Suzuki et al., 2011) via the Maximum Likelihood method with 1,000 bootstrap replicates. The resulting phylogeny (**Figure 1**) classified the SOD proteins into three functional categories: Mn-SOD, Fe-SOD, and Cu/Zn-SOD, consistent with established classification systems (Apel and Hirt, 2004). The iTOL v5 interface (https://itol.embl.de/) was used for tree annotation and topological refinement (Sasaki-Sekimoto et al., 2005). The structural features of *LbaSOD*s were analyzed using the Gene Structure Display Server 2.0 (http://gsds.gao-lab.org/, accessed 25 January 2024) (Devoto and Turner, 2003), with exon-intron configurations visualized schematically, including coding regions represented as green boxes and introns as connecting lines (**Figure 2b**). Protein motif analysis was conducted using the MEME Suite web service (https://meme-suite.org/meme/, accessed 25 January 2024) (Miura and Tada, 2014), which was configured to detect 10 distinct motifs ranging from 15 to 50 residues in length. Ten conserved motifs were identified across all *LbaSODs*, with sequence logos illustrating residue conservation patterns (**Figure 3**). These structural elements were mapped onto a phylogenetic framework to investigate the evolutionary diversification of domain architecture.

### 2.4 Chromosomal localization and synteny analyses of *LbaSOD*s

The chromosomal locations of the identified *LbaSOD* genes were determined using genomic coordinates from the annotation file and visualized using the online tool MG2C v2.1 (MapGene2Chromosome). Genes located on unanchored scaffolds were excluded from this analysis. To investigate evolutionary relationships and syntenic conservation, the protein sequences encoded by *LbaSODs* were used as queries in BLASTP searches against the proteomes of *S. lycopersicum*, *S. tuberosum*, and *A. thaliana*. The identified homologous gene pairs and their corresponding genomic positions were analyzed using TBtools (Chen et al., 2020) to detect and visualize collinear regions using the Dual Synteny Plotter utility.

### 2.5 Prediction of *Cis*-elements prediction in *LbaSOD* promoters

To investigate the potential regulatory mechanisms of *LbaSOD*s, promoter sequences comprising 2,000 bp upstream regions of the transcription start site for each *LbaSOD* were retrieved from the wolfberry genome using TBtools. Putative *cis*-acting elements within these promoter regions were then predicted by submitting the sequences to the PlantCARE database (https://bioinformatics.psb.ugent.be/webtools/plantcare/html/, accessed 20 January 2024). The distribution and types of identified *cis*-elements were summarized graphically using TBtools for visualization.

### 2.6 Expression analysis of *LbaSOD*s in different tissues and at various fruit developmental stages of wolfberry

Wolfberry plants were grown in a controlled growth chamber. One-month-old soil-grown seedlings were used to examine the expression patterns of *LbaSOD*s in various tissues. Samples, including roots, stems, leaves, flowers, and fruits, were collected at five distinct developmental stages for subsequent RNA extraction. The fruit developmental stages were defined as follows: including S1 (12 DAF), S2 (19 DAF), S3 (25 DAF), S4 (30 DAF), and S5 (37 DAF), where DAF denotes days after full bloom. All harvested samples were immediately frozen in liquid nitrogen and stored at -80 °C until further processing. Total RNA was isolated using the TRNzol Universal Total RNA Isolation Kit (Tiangen, Beijing, China), following the manufacturer’s instructions. First-strand cDNA was synthesized from 1 µg of total RNA using the HiScript II 1^st^ Strand cDNA Synthesis Kit (Vazyme, Nanjing, China). Quantitative real-time PCR (qRT-PCR) was performed on a QuantStudio 5 system (Applied Biosystems, USA) using ChamQ Universal SYBR qPCR Master Mix (Vazyme) with three independent biological and three technical replicates. The *LbaACTIN* served as the internal control. Primer amplification efficiency was evaluated using standard curves generated from serial cDNA dilutions ranging from 95% to 105%. Melting curve analysis confirmed a single sharp peak for each amplicon, indicating high amplification specificity. *LbaACTIN* was selected as the internal reference based on its stable expression across all tested tissues, developmental stages, and treatment conditions in preliminary assays. Relative gene expression levels were calculated using the 2^-ΔΔCt^ method (Livak and Schmittgen, 2001). Gene-specific primers (**Supplementary Table 3**) were designed based on the coding sequences (CDS) of target genes. Statistical analyses were conducted using Data Processing System software (version 18.0). Data normality and homogeneity of variances were assessed using Shapiro-Wilk’s and Levene’s tests, respectively, before analysis of variance (ANOVA). One-way ANOVA followed by Duncan’s multiple range test was used to determine the significant differences (*p* < 0.05). This statistical workflow was consistently applied to all qRT-PCR datasets.

### 2.7 Plant materials and treatments for qRT-PCR analysis

Wolfberry plants were grown in a greenhouse at 25 °C, under a light intensity of 800 μmol·m^–^²·s^–^¹, with a 16-hour light/8-hour dark photoperiod and 60–65% relative humidity. One-month-old soil-grown seedlings were used for abiotic stress and phytohormone treatment analyses. For abiotic stress treatments, uniformly grown seedlings were exposed to cold stress at 4 °C, 20% (w/v) PEG-6000 solution to simulate drought stress, and 200 mmol/L NaCl solution to simulate salt stress, concentrations widely adopted in previous studies on *Solanaceous* plants to effectively activate downstream signaling pathways (Wang et al., 2005). For phytohormone treatments, seedlings were treated with 100 μM solutions of IAA, GA, ABA, SA, and MeJA, as described by Xu et al. (2022). The leaves from all treatment groups were collected at 0, 1, 3, 6, 9, 12, and 24 hours post-treatment, immediately frozen in liquid nitrogen, and stored at −80 °C until RNA extraction. *LbaACTIN* was used as the internal control. Relative gene expression levels were calculated using the 2^−ΔΔCt^ method. Total RNA was extracted and qRT-PCR was performed as described in Section 2.6. All gene-specific primers were designed based on the CDS sequences of target genes (**Supplementary Table 3**).

## 3 Results

### 3.1 Identification and characteristics of *LbaSOD*s

A comprehensive genome-wide survey identified 10 non-redundant SOD genes​ in the wolfberry genome, designated *LbaSOD1* to *LbaSOD10* based on their chromosomal locations (**Supplementary Table 1**). The CDS of the SOD genes ranged from 465 to 1182 bp and encoded proteins of 154–393 amino acids in length. The predicted molecular weights of the proteins varied from 15.5 to 45.2 kDa, whereas the theoretical isoelectric points ranged from 5.2 to 9.1. All *LbaSODs* exhibited negative grand average hydropathicity (GRAVY) values, indicating their hydrophilic nature.

### 3.2 Phylogenetic relationships and classification of *LbaSOD*s

To determine the phylogenetic relationships and categories of *LbaSOD*s, an evolutionary tree was generated using SOD protein sequences from *L. barbarum*, *S. lycopersicum*, *S. tuberosum* (potato), *O. sativa* (rice), and *A. thaliana* (**Figure 1**). Based on the evolutionary conservation of *AtSOD*s, the *LbaSOD*s were categorized into three subfamilies: the Fe-SOD and Cu/Zn-SOD subfamilies, with four *LbaSOD*s each, and the Mn-SOD subfamily, with only two genes. All *LbaSOD*s demonstrated robust phylogenetic clustering with strong bootstrap support, confirming reliable evolutionary relationships among the subfamilies.

### 3.3 Analysis of gene structure and conserved motifs in *LbaSOD*s

To gain further insights into the structural diversity and evolutionary conservation of *LbaSOD*s, we analyzed their gene structures and conserved protein motifs and constructed a phylogenetic tree (**Figure 2a**) using full-length *LbaSODs* to provide an evolutionary framework for subsequent analysis. Each member of the Fe-SOD, Mn-SOD, and Cu/Zn-SOD subfamilies exhibited remarkably similar gene structures and motif compositions, strongly supporting their phylogenetic relationships. The examination of the organization of the *LbaSOD* exons and introns also indicated similarities in the number and length of genes in the same subfamily. For instance, all *LbaCu/Zn-SODs* and *LbaMn-SODs* contained six introns, whereas the *LbaFe-SODs* possessed 7–10 introns (**Figure 2b**). This high degree of structural conservation within subfamilies suggests a strong evolutionary constraint on the architecture of the *LbaSOD*s.

We also analyzed the conserved functional domains and MEME-predicted conserved motifs of LbaSOD proteins. Visualization of Pfam-conserved superfamily domains (**Figure 3B**) revealed distinct domain composition patterns among the Fe-SOD, Mn-SOD, and Cu/Zn-SOD subfamilies. The distribution of conserved motifs is illustrated in **Figure 3c**, and the sequence logos of the ten identified motifs are shown in **Figure 3d**. Consistent with the domain analysis results, proteins within the same phylogenetic subfamily shared identical motif combinations. Specifically, motifs 1, 2, 3, 6, and 10 were universally present in all Mn-SOD proteins, whereas motifs 1, 2, 6, and 9 were commonly identified and conserved among all Fe-SOD proteins. In contrast, motif 4 was uniquely conserved among all Cu/Zn-SODs. The metal-binding and catalytic functional domains coincided with these conserved motifs, highlighting their essential roles in SOD.

Overall, the combined analysis of phylogeny, conserved protein domains, and peptide motifs supported the subfamily classification of LbaSODs and demonstrated their functional conservation across the subfamilies.

### 3.4 Chromosomal distribution of *LbaSOD*s

The chromosomal distribution of the 10 identified *LbaSOD*s was analyzed across the 12 wolfberry chromosomes, revealing an uneven distribution of genes, with each of chromosomes 9 and 10 harboring two *LbaSOD*s. Eight chromosomes, including Chr1, 2, 3, 4, 5, 6, 7, and 8, carried a single *LbaSOD* each, whereas no SOD genes were mapped to Chr 11 and 12 (**Supplementary Figure 1**). This irregular distribution pattern suggests that *LbaSOD*s are not randomly localized in the genome. Furthermore, no significant correlation was observed between chromosome size and the number of *LbaSOD*s.

### 3.5 Chromosomal location and synteny analysis of *LbaSOD*s

To elucidate the evolutionary history of *LbaSOD*s, we performed intra-species synteny analysis, revealing three paralogous gene pairs located in three syntenic blocks within the wolfberry genome (**Supplementary Figure 2a**). These segmental duplication events involved the pairing of *LbaSOD5* with *LbaSOD6*, *LbaSOD4* with *LbaSOD8*, and *LbaSOD7* with *LbaSOD1*, indicating that this gene family expanded mainly via interchromosomal duplication. Further comparative syntenic analysis conducted identified 6, 4, and 1 homologous SOD gene pairs between wolfberry and tomato, potato, and *Arabidopsis*, respectively (**Supplementary Figure 2b**), suggesting a closer evolutionary relationship between SOD genes in wolfberry and tomato.

### 3.6 *Cis*-element analysis of the *LbaSOD*s

**Figure 4:**
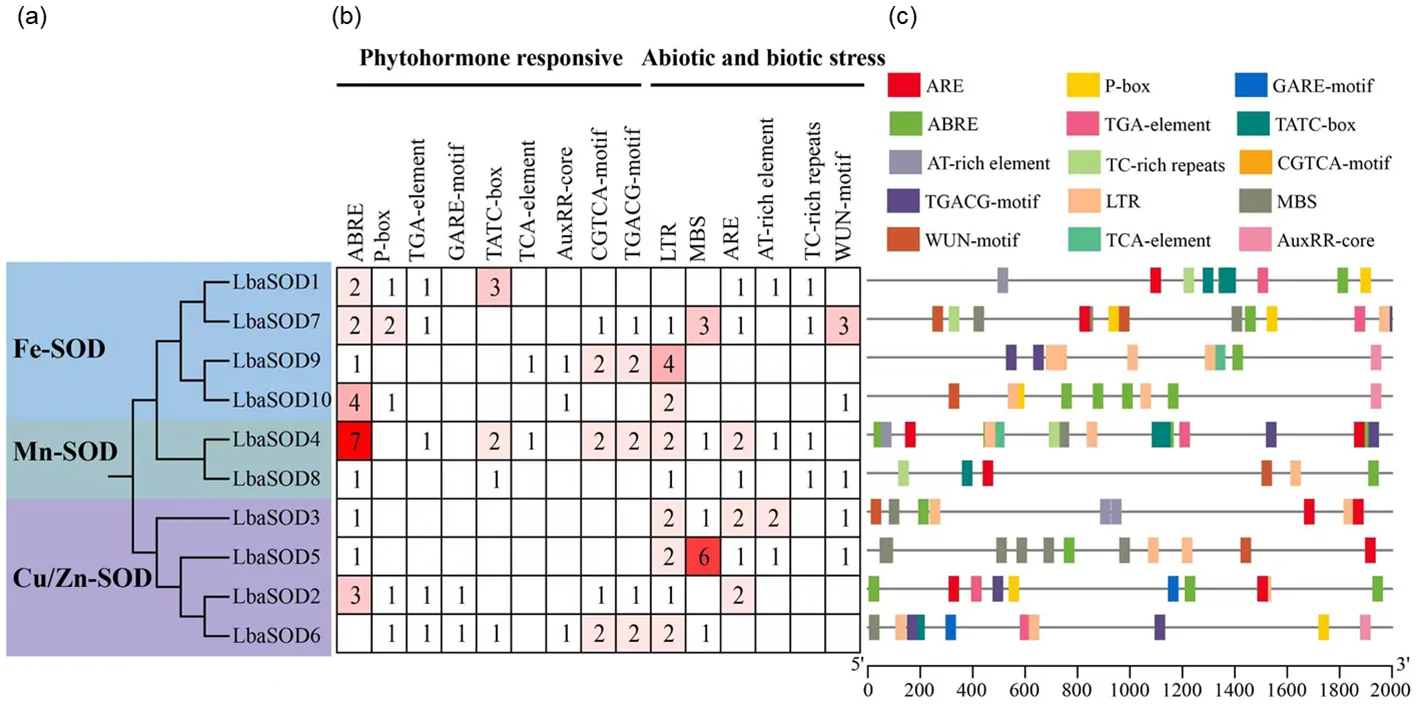
Investigation of cis-acting element numbers in *LbaSOD*s. **(a)** Phylogenetic tree of *LbaSODs*. The display uses different background colors to distinguish the subgroups. **(b)** The different colors and numbers of grids indicate the number of different promoter elements in these *LbaSOD*s. **(c)** The different blocks represent the different types of cis-acting elements and their locations in each *LbaSOD* gene.

One hundred and ten *cis*-regulatory elements were identified in the *LbaSOD* promoters and classified into two primary functional subgroups: phytohormone responsiveness and abiotic and biotic stress responses (**Figure 4**; **Supplementary Table 2**). The abiotic and biotic stress response subgroup included low-temperature response (LTR), drought inducibility (MBS), anaerobic induction (ARE), AT-rich element (related to stress response), TC-rich repeats (defense and stress responsiveness), and WUN-motif (wound response) elements. Of these, LTR elements were the majority, with 17 elements, and were present in nearly all the *LbaSOD* promoters. Conversely, the phytohormone-responsive elements associated with hormone signaling, included ABA-responsive element (ABRE), P-box, and GARE-motif (gibberellin response), TGA and TGACG-motif (jasmonic acid [JA] response), TCA (SA response), TATC-box (gibberellin response), AuxRR-core (auxin response), and CGTCA-motif (JA response). Overall, ABRE elements were the most abundant, with 22 copies identified in almost every *LbaSOD* promoter. The presence of ABRE and LTR elements in all the *LbaSOD*s suggests that they are central to ABA signaling and cold stress adaptation in wolfberry, while the abundance of these stress- and hormone-related cis-elements strongly implies that the expression of the *LbaSOD*s is under complex regulatory control and that they play crucial roles in the adaptation of wolfberry to various environmental stresses and hormonal cues.

### 3.7 Expression patterns of *LbaSOD*s in different organs

**Figure 5:**
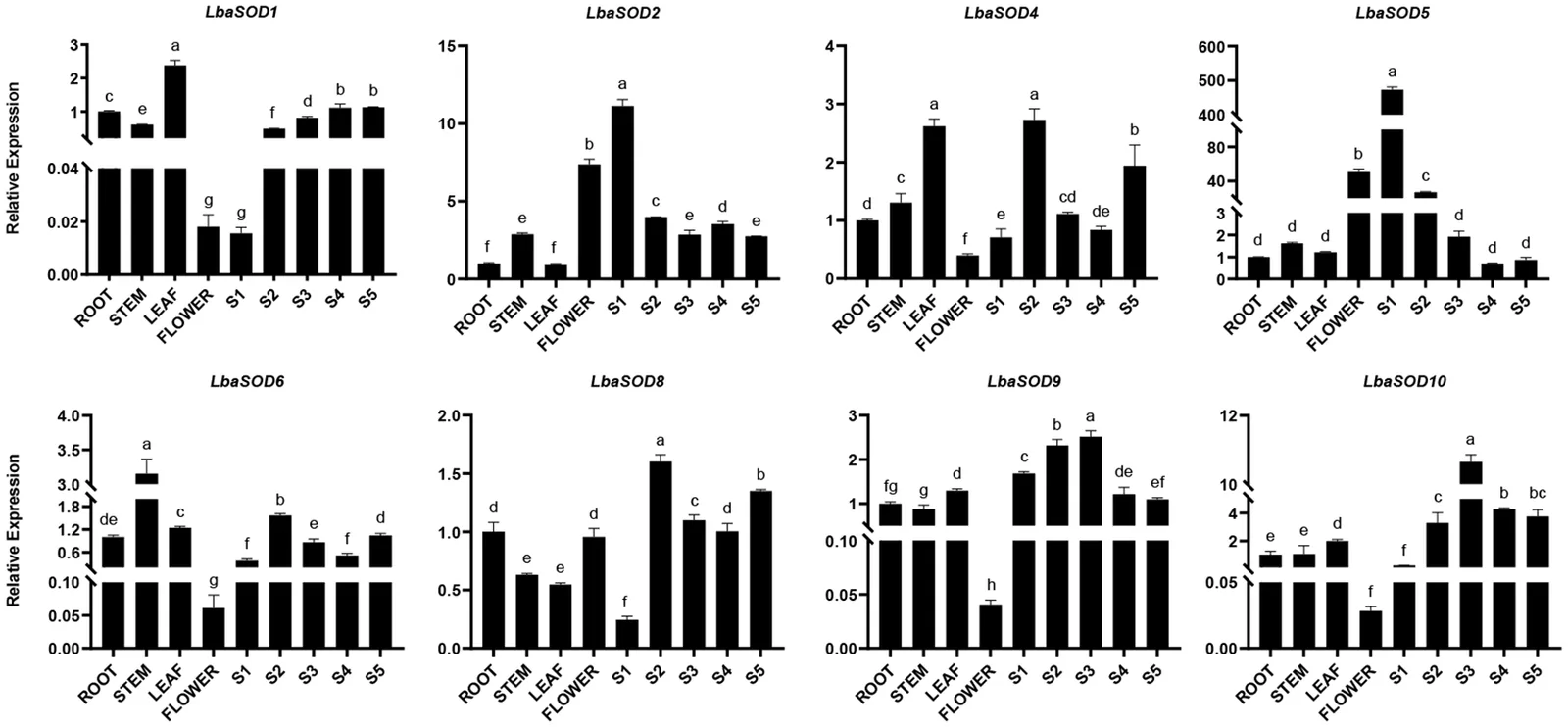
Relative expression levels of eight *LbaSOD*s in different wolfberry tissues. Expression level analysis of target genes across fruit developmental stages using *actin* as the internal reference. The x-axis represents root, stem, leaf, flower, and five key fruit development time points: 12, 19, 25, 30, and 37 DAF. The y-axis denotes the relative expression levels of the genes. Data are shown as the mean ± standard deviation (SD) with three biological replicates (n = 3). Different letters above bars indicate significant differences in mean values in different organs (*p* < 0.05, one-way ANOVA analysis [Duncan’s multiple range test]).

The expression profiles revealed distinct temporal and spatial dynamics for different *LbaSOD* members. For instance, the expression of *LbaSOD1* and *LbaSOD4* was significantly decreased in flowers and at the early fruit developmental stage (S1), but increased from S2 to S5 (**Figure 5**), whereas the expression of *LbaSOD2* and *LbaSOD5* increased rapidly in flowers and during early fruit development, peaking at S1 but gradually declining thereafter, suggesting their important roles in initial fruit development. However, the expression of *LbaSOD6*, *LbaSOD9*, and *LbaSOD10* was lowest in flowers but markedly increased across all subsequent fruit developmental stages, implying their potential involvement in fruit maturation and ripening processes. Furthermore, *LbaSOD8* reached its expression minimum at the S1 stage, which may imply its distinct regulatory pattern or its reduced requirement during the initial fruit development. These results indicate that *LbaSOD*s possess diverse and specific expression patterns across different tissues and fruit development stages and suggest their crucial and stage-specific roles in fruit development and stress responses in wolfberry.

### 3.8 Expression patterns of *LbaSOD*s under abiotic stress

**Figure 6:**
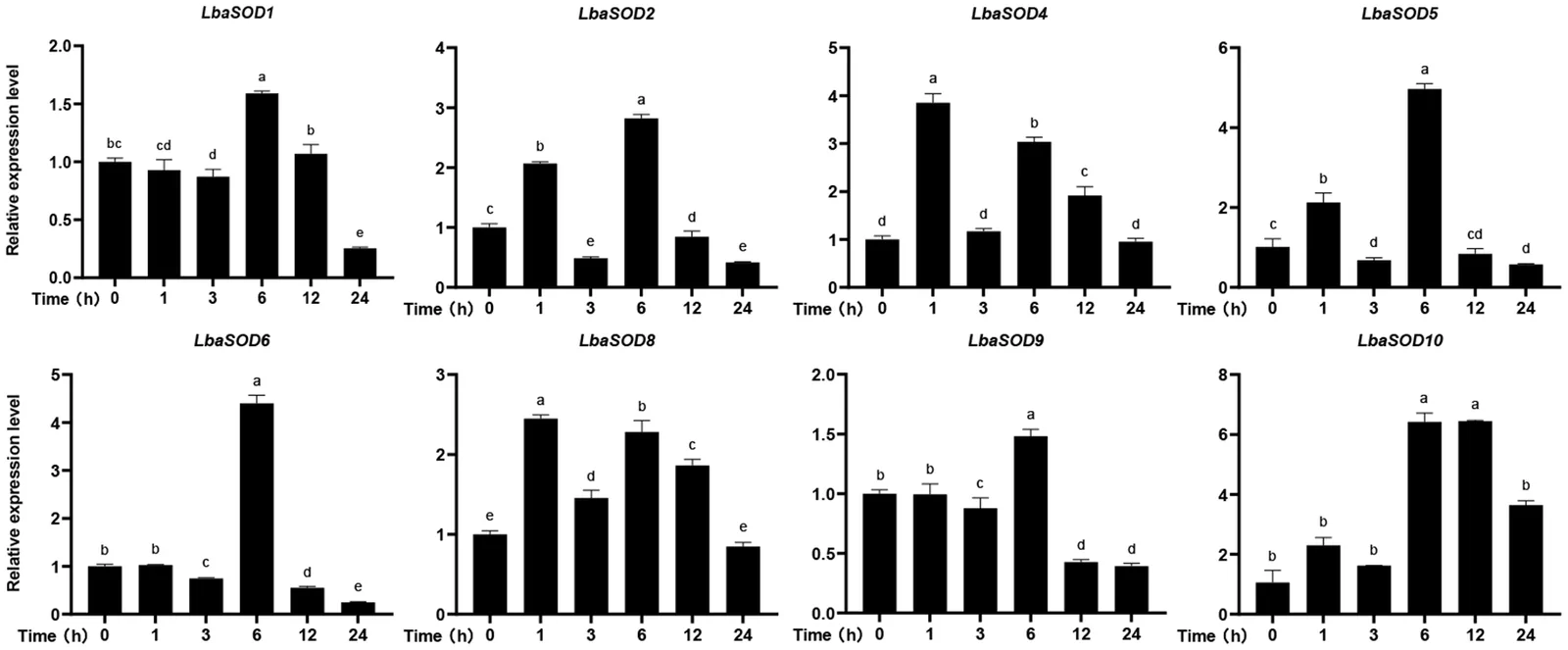
Relative expression levels of eight *LbaSOD*s in response to cold stress. Plants were subjected to cold treatment, and their leaves were collected after 0, 1, 3, 6, 12, and 24 hours, respectively. The x-axis represents cold treatment time, and the y-axis denotes the relative expression levels normalized to the *actin* gene. Data are presented as the mean ± SD of three biological replicates (n = 3). Significant differences between treatments are indicated by different letters (*p* < 0.05, one-way ANOVA [Duncan’s multiple range test]).

To investigate the expression patterns of *LbaSOD*s under abiotic stress conditions, we performed qRT-PCR analysis on eight detectable *LbaSOD*s under cold, salt, and drought treatments. The expression of *LbaSOD3* and *LbaSOD7* was not detected in the seedlings under the tested conditions and was therefore excluded from the analysis. The results demonstrated distinct temporal expression dynamics among different SOD subfamilies. For example, under cold stress, members of the Fe-SOD subfamily, including *LbaSOD1*, *LbaSOD9*, and *LbaSOD10*, and one Cu/Zn-SOD gene, of *LbaSOD6* showed relatively stable expression during the early phase, peaking at 6 hours, and subsequently declining thereafter (**Figure 6**).

**Figure 7:**
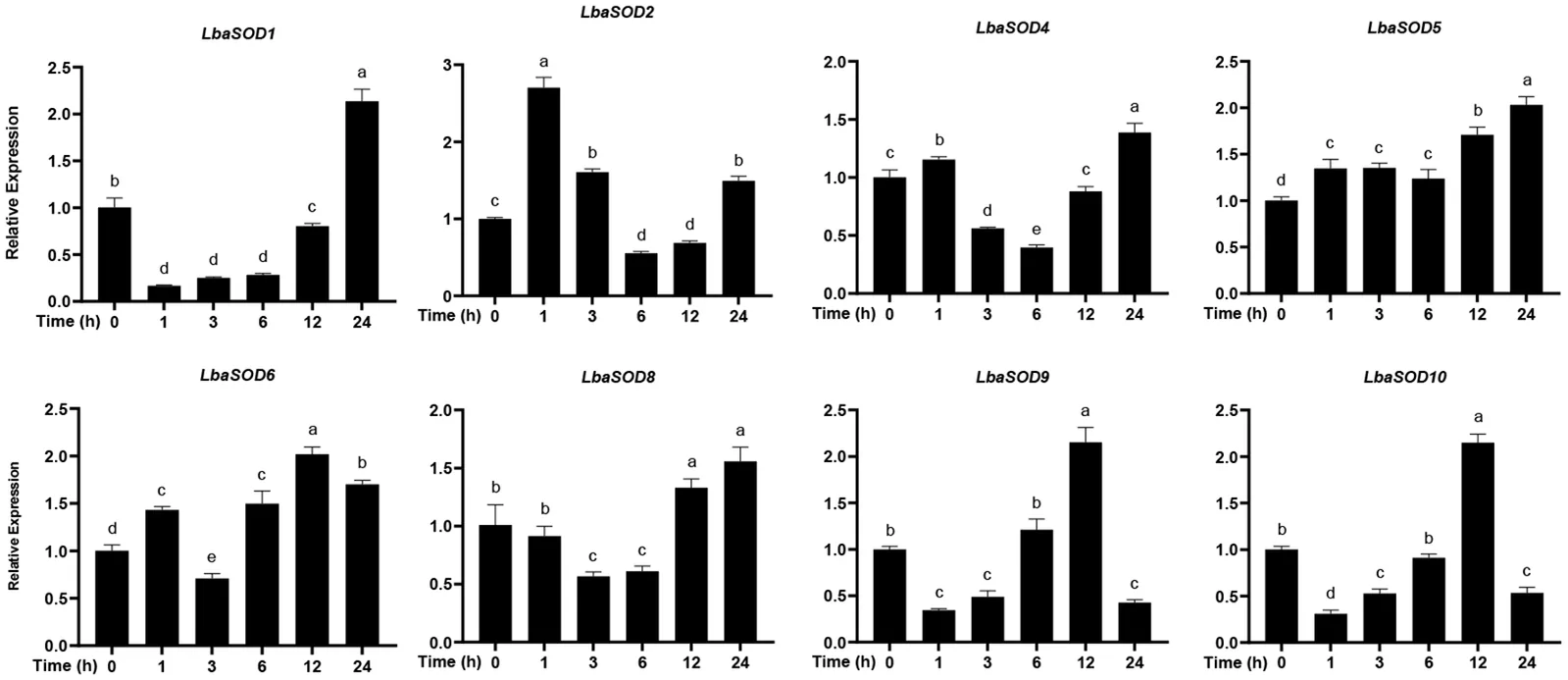
Relative expression levels of *LbaSOD*s in response to salt stress. Plants were subjected to salt treatment, and their leaves were collected after 0, 1, 3, 6, 12, and 24 hours, respectively. The x-axis represents salt treatment time, and the y-axis denotes the relative expression levels normalized to the *actin* gene. Data are presented as the mean ± SD of three biological replicates (n = 3). Significant differences between treatments are indicated by different letters (*p* < 0.05, one-way ANOVA [Duncan’s multiple range test]).

In response to salt stress, the Cu/Zn-SOD genes, including *LbaSOD2*, *LbaSOD5*, and *LbaSOD6* and the Mn-SOD gene of *LbaSOD4* displayed a fluctuating expression pattern, with an initial increase, followed by a decrease, and then a rebound, reaching approximately 1.4-fold of the baseline. In contrast, the Fe-SOD genes, including *LbaSOD1*, *LbaSOD9*, and *LbaSOD10*, were significantly induced after an initial downregulation, ultimately reaching approximately 2-fold of the initial level (**Figure 7**).

**Figure 8:**
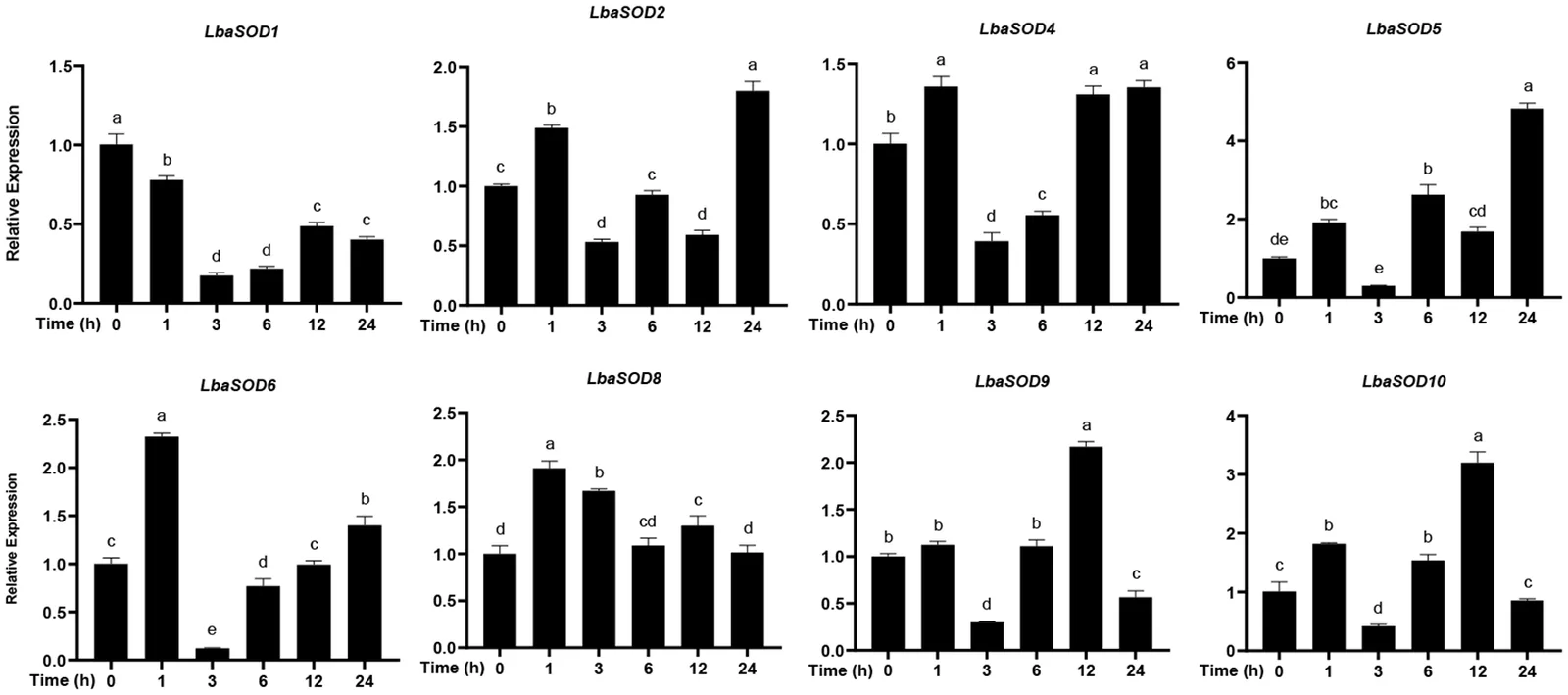
Relative expression levels of eight *LbaSOD*s in response to drought stress. Plants were subjected to drought treatment, and their leaves were collected after 0, 1, 3, 6, 12, and 24 hours, respectively. The x-axis represents the drought treatment time, and the y-axis denotes the relative expression levels normalized to the *actin* gene. Data are presented as the mean ± SD of three biological replicates (n = 3). Significant differences between treatments are indicated by different letters (*p* < 0.05, one-way ANOVA [Duncan’s multiple range test]). Different lowercase letters indicate significant differences (p < 0.05), while identical letters indicate no significant difference.

Under drought conditions, most *LbaSOD*s were initially upregulated before being downregulated. Notably, Fe-SOD genes, including *LbaSOD1*, *LbaSOD9*, and *LbaSOD10*, were strongly suppressed at 24 hours, declining to nearly half of their original expression. Except for *LbaSOD8*, which did not show a pronounced decrease, all other SOD genes reached their expression minima at 3 hours post-treatment (**Figure 8**). These results indicate that *LbaSOD*s in wolfberry respond to abiotic stresses in both a subfamily- and gene-specific manner, underscoring functional divergence and coordination within the antioxidant defense system.

### 3.9 Expression patterns of *LbaSOD*s under phytohormones

**Figure 9:**
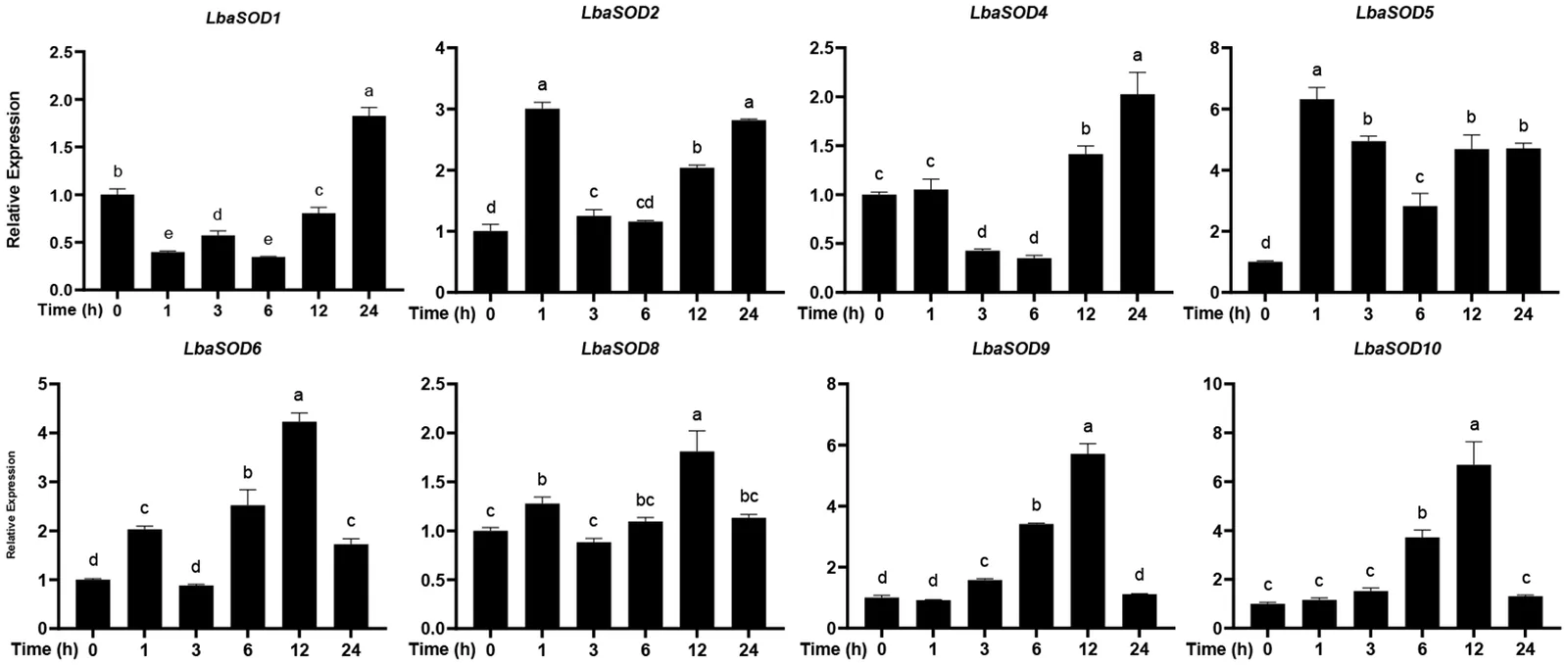
Relative expression levels of *LbaSOD*s in response to IAA. Plants were exposed to IAA for 0, 1, 3, 6, 9, 12, and 24 hours. The x-axis represents the time after IAA treatment, and the y-axis denotes the relative expression levels normalized to the *actin* gene. Data are presented as the mean ± SD of three biological replicates (n = 3). Significant differences between treatments are indicated by different letters (*p* < 0.05, one-way ANOVA [Duncan’s multiple range test]).

To further explore the potential roles of *LbaSOD*s in hormone-mediated stress responses, we analyzed their expression profiles under treatments with GA, IAA, SA, JA, and ABA using qRT-PCR. The results revealed complex and hormone-specific regulatory patterns among different SOD subfamilies. Under exposure to IAA, *LbaSOD1*, *LbaSOD4*, and *LbaSOD8* were initially downregulated and then induced, with expression levels of *LbaSOD1* and *LbaSOD4* decreasing to approximately 0.25-fold compared to the control before rising to approximately 2-fold. In contrast, the expression of *LbaSOD2*, *LbaSOD5*, and *LbaSOD6* increased, followed by a decline to steady state and a subsequent rebound. *LbaSOD9* and *LbaSOD10* were also initially induced with a slight decline at 9 hours, and a sharp increase reaching 6-7-fold of the baseline (**Figure 9**).

**Figure 10:**
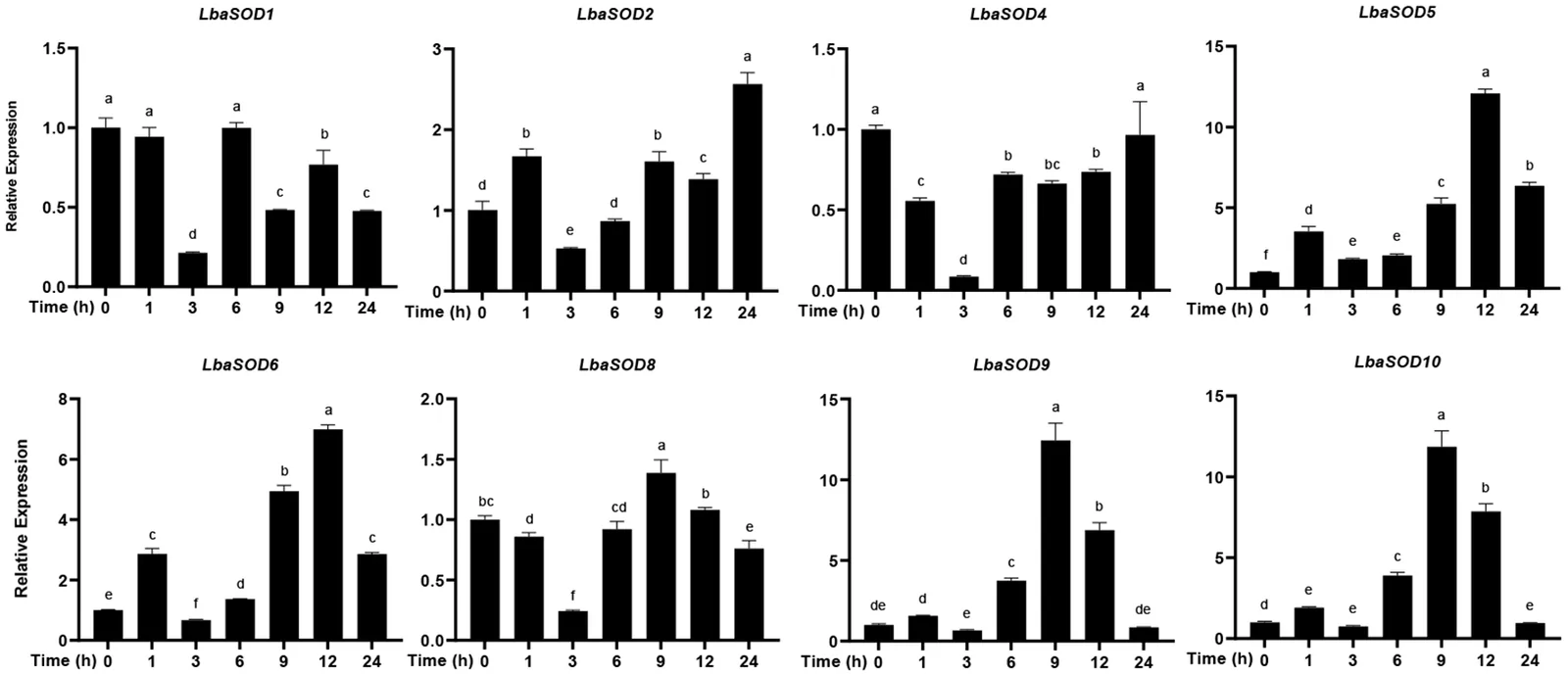
Relative expression levels of *LbaSOD*s in response to GA. Plants were exposed to GA treatment for 0, 1, 3, 6, 9, 12, and 24 hours. The x-axis represents time after GA treatment, and the y-axis denotes relative expression levels normalized to the *actin* gene. Data are presented as the mean ± SD of three biological replicates (n = 3). Significant differences between treatments are indicated by different letters (*p* < 0.05, one-way ANOVA [Duncan’s multiple range test]).

Following GA exposure, *LbaSOD2*, *LbaSOD5*, *LbaSOD6*, *LbaSOD9*, and *LbaSOD10* exhibited a multiphasic expression pattern characterized by a slight initial increase, followed by a decline, substantial upregulation, and subsequent decrease, whereas *LbaSOD1*, *LbaSOD4*, and *LbaSOD8* were initially downregulated, followed by recovery and stabilization (**Figure 10**).

**Figure 11:**
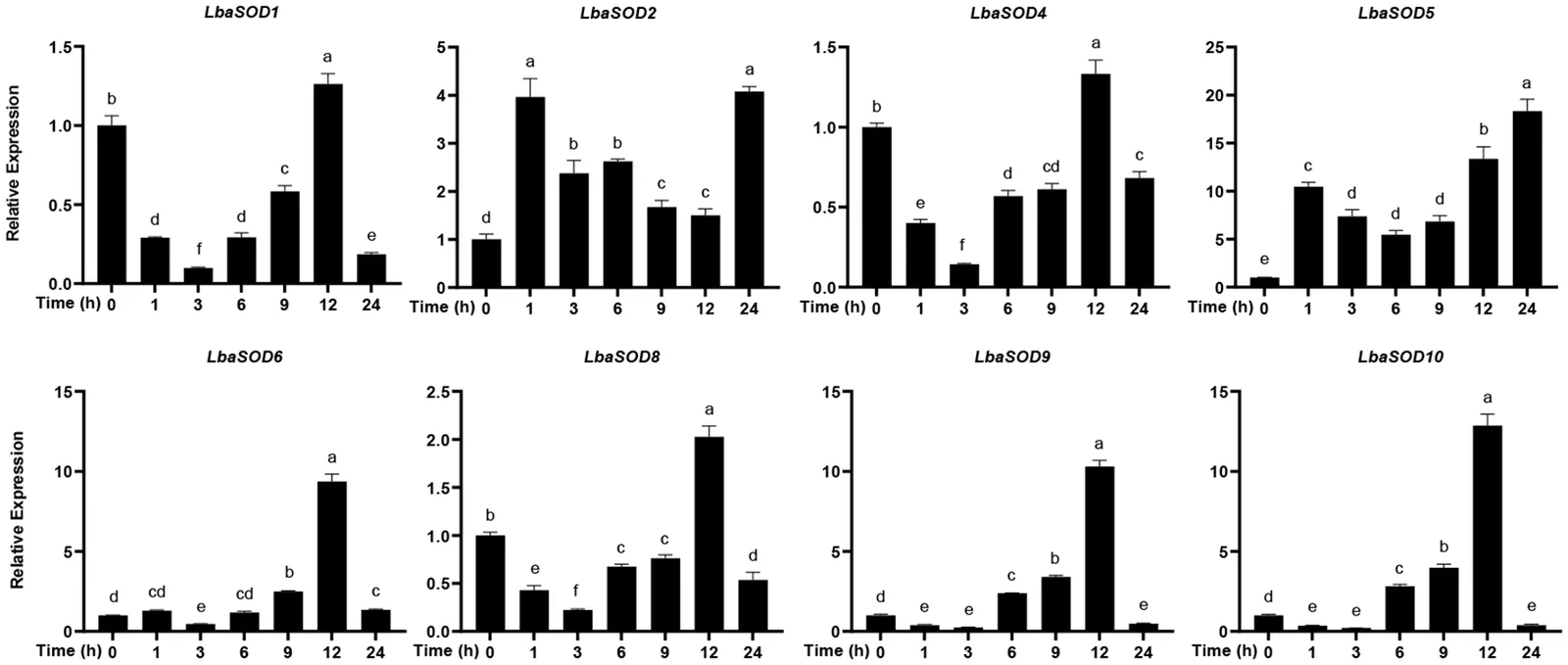
Relative expression levels of *LbaSOD*s in response to SA after 0, 1, 3, 6, 9, 12, and 24 hours of exposure. The x-axis represents the time after SA treatment, and the y-axis denotes the relative expression levels normalized to the *actin* gene. Data are presented as the mean ± SD of three biological replicates (n = 3). Significant differences between treatments are indicated by different letters (*p* < 0.05, one-way ANOVA [Duncan’s multiple range test]).

*LbaSOD1*, *LbaSOD9*, *LbaSOD10*, *LbaSOD4*, *LbaSOD8*, and *LbaSOD6* exhibited a conserved expression pattern featuring significant initial suppression, followed by a rapid and strong upregulation and subsequent decline in response to SA. In contrast, the expression of *LbaSOD9* and *LbaSOD10* peaked at over 10-fold of the control, whereas that of *LbaSOD6* reached nearly a 10-fold increase. Additionally, *LbaSOD2* and *LbaSOD5* showed a distinct response to SA, characterized by a rapid initial increase, a slight decline, and then a second sharp rise, thereby maintaining high expression levels throughout the treatment period. Their expression also peaked at 24 hours, reaching approximately 4- and 18-fold of the control, respectively (**Figure 11**).

**Figure 12:**
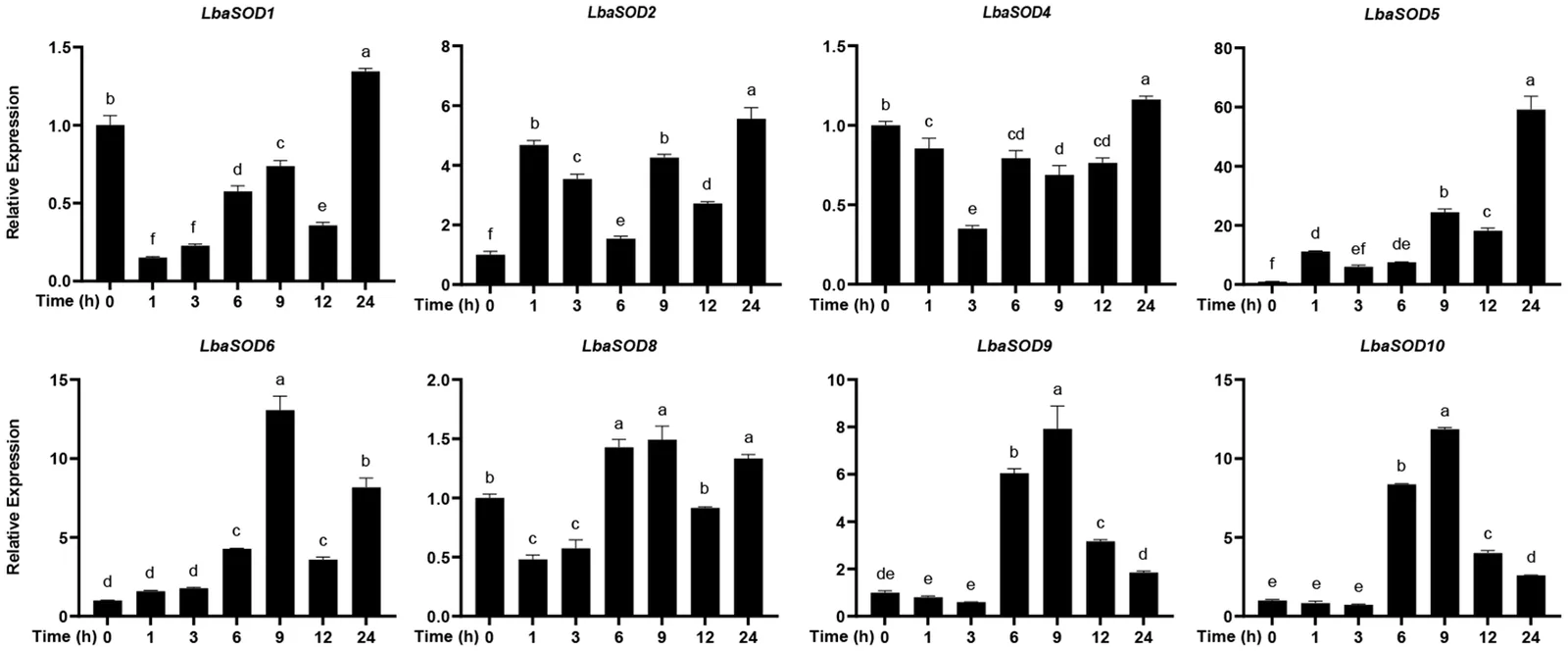
Relative expression levels of *LbaSOD*s in response to MeJA. MeJA exposure for 0, 1, 3, 6, 9, 12, and 24 hours. The x-axis represents time after MeJA treatment, and the y-axis denotes relative expression levels normalized to the *actin* gene. Data are presented as the mean ± SD of three biological replicates (n = 3). Significant differences between treatments are indicated by different letters (*p* < 0.05, one-way ANOVA [Duncan’s multiple range test]).

Under MeJA treatment, the *LbaSOD9* and *LbaSOD10* genes were transiently suppressed before rising sharply to 8- and 12-fold of the control, respectively, and then declined to levels slightly above baseline. *LbaSOD2*, *LbaSOD5*, and *LbaSOD6* maintained high expression levels throughout the treatment, despite some fluctuations. For example, the expression of *LbaSOD5* dramatically increased to approximately 60-fold of the control at 24 hours. Conversely, *LbaSOD1*, *LbaSOD4*, and *LbaSOD8* were initially suppressed to 0.2-0.5-fold before recovering to 1.2-1.4-fold of the initial level (**Figure 12**). These results suggest that *LbaSOD*s are involved in intricate hormone-responsive networks and exhibit hormone- and subfamily-specific expression patterns, highlighting their potential roles in phytohormone-mediated antioxidant defense mechanisms.

**Figure 13:**
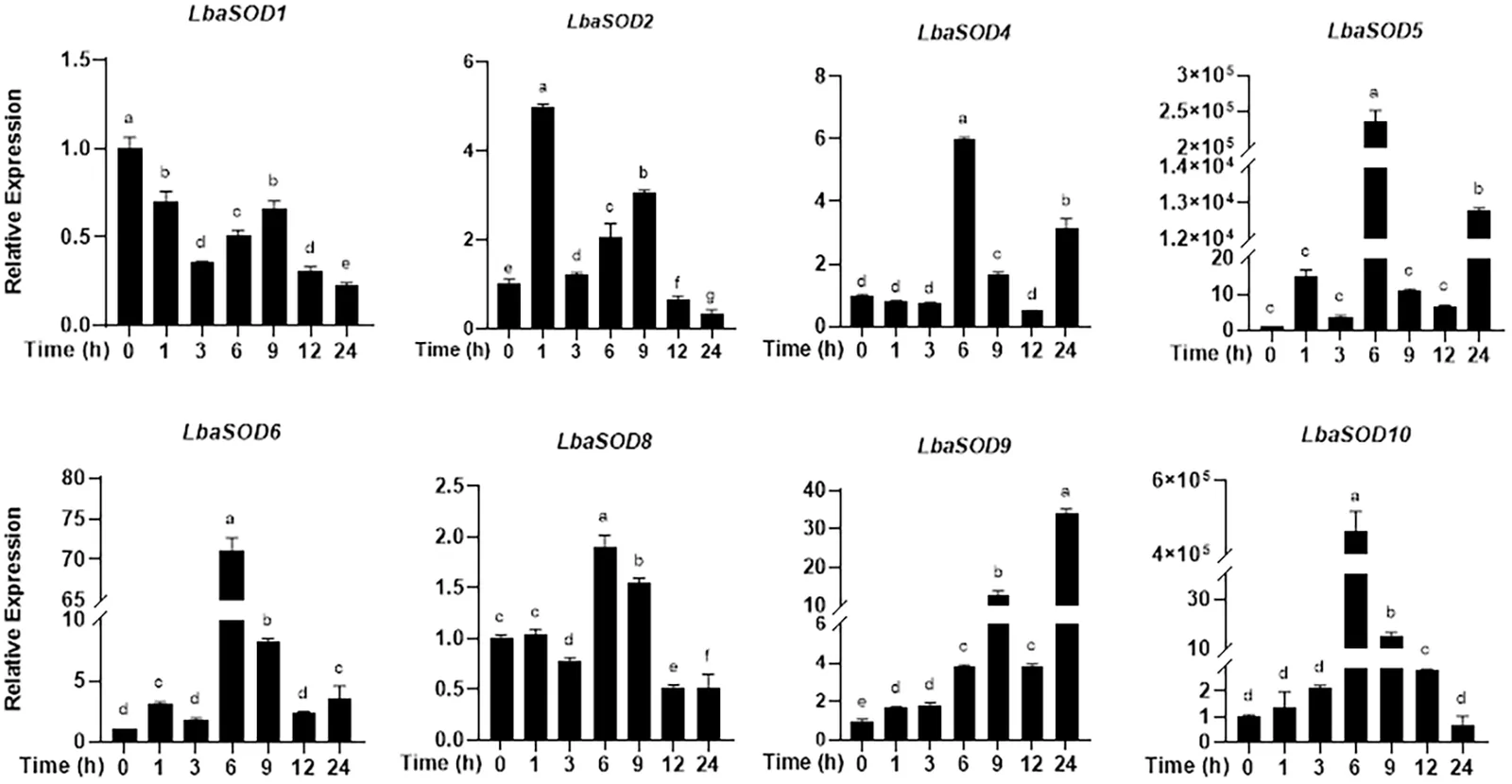
ABA-induced expression analysis of eight members of the SOD gene family using qRT-PCR. Relative expression levels of *LbaSODs* in response to ABA treatment are shown. Plants were treated with ABA, and leaf samples were collected at 0, 1, 3, 6, 9, 12, and 24 hours post-treatment. The x-axis represents the time after ABA treatment, and the y-axis indicates the relative expression levels normalized to the *actin* gene. Data are presented as the mean ± SD of three biological replicates (n = 3). Significant differences between treatments are denoted by different letters (*p* < 0.05, one-way ANOVA, followed by Duncan’s multiple range test).

In response to ABA treatment, all eight detectable *LbaSOD*s exhibited significant changes in expression, with distinct temporal dynamics (**Figure 13**). *LbaSOD4*, *LbaSOD5*, *LbaSOD6*, *LbaSOD8*, and *LbaSOD10* reached their peak expression levels at 6 hours post-treatment. Among these, *LbaSOD6* was upregulated approximately 70-fold relative to the control, whereas *LbaSOD5* and *LbaSOD10* showed extremely strong induction, with approximately 2.5 × 10^5^-fold and 6 × 10^5^-fold increases, respectively. After their peaks, the expression of *LbaSOD4*, *LbaSOD5*, and *LbaSOD6* declined but subsequently increased again at 24 hours, indicating a biphasic regulatory pattern. *LbaSOD9* expression peaked at 24 hours with an approximately 35-fold increase. *LbaSOD2* exhibited the earliest response, reaching its maximum at 1 hour with an approximately 5-fold induction. In contrast, *LbaSOD1* expression was suppressed by ABA treatment, declining to a minimum at 3 hours and fluctuating thereafter, ultimately reaching its lowest level at 24 hours (approximately 20% of the control level). These results demonstrate that ABA acts as a potent and gene-specific transcriptional regulator of the *LbaSOD* family, eliciting both strong transcriptional activation of specific members and selective suppression of others.

## 4 Discussion

The SOD gene family plays an indispensable role in plant growth, development, and adaptation to environmental stresses by maintaining the cellular redox homeostasis. This study presents the first comprehensive genome-wide analysis of the *LbaSOD* family in wolfberry, an economically important medicinal species of the *Solanaceae* family. Our integrated approach, which combined phylogenetic analysis, structural characterization, *cis*-element prediction, and systematic expression profiling across various tissues, abiotic stresses, and five phytohormones, revealed a regulatory architecture that is both evolutionarily conserved and functionally diverse.

The identification of 10 *LbaSOD*s is consistent with gene counts reported in closely related *Solanaceae* species, such as tomato (9) and potato (8) (Feng et al., 2016; Rudić et al., 2022), but is lower than that in species that have undergone recent whole-genome duplication, such as *Brassica napus* (18) and soybean (13) (Verma et al., 2019; Lu et al., 2020). The three subfamilies, Fe-SOD, Mn-SOD, and Cu/Zn-SOD, were strongly supported by phylogenetic analysis, conserved domain architecture, gene structure, and motif compositions. The characteristic metal-binding residues are highly conserved across all members, underscoring functional fidelity throughout evolution (Miller, 2012). Segmental duplication, rather than tandem duplication, was the primary mechanism underlying *LbaSOD* gene family expansion, as evidenced by the presence of three segmentally duplicated paralogous pairs. This pattern mirrors the duplication dynamics reported for SOD gene families in other *Solanaceae* species (Feng et al., 2016) and is consistent with the predominance of segmental duplication in driving gene family expansion within this lineage (Cannon et al., 2004). Furthermore, the greater number of syntenic SOD gene pairs identified between wolfberry and tomato further supports their close evolutionary relationships (Tang et al., 2008; The Tomato Genome Consortium, 2012).

The *cis*-element landscape of *LbaSOD* promoters revealed a strong enrichment of stress- and hormone-responsive motifs, with ABRE and LTR elements being the most abundant. This promoter architecture suggests that *LbaSOD* expression is primarily regulated by ABA signaling and cold stress pathways (Yamaguchi-Shinozaki and Shinozaki, 2005; Fujita et al., 2011). Our expression data largely supported these predictions: cold stress induced several Fe-SOD members, and ABA triggered exceptionally strong transcriptional responses in multiple *LbaSOD*s. However, the relationship between *cis*-element abundance and expression output was not uniformly predictive; the presence of ABRE motifs was necessary but not sufficient to determine the magnitude, kinetics, or even the direction (induction vs. suppression) of ABA responsiveness, a limitation exemplified by the contrasting behavior of *LbaSOD10* (massive induction) and *LbaSOD1* (sustained suppression). This suggests that additional regulatory mechanisms, including motif context, chromatin accessibility, and transcription factor availability, may shape the ultimate transcriptional output (Nakashima and Yamaguchi-Shinozaki, 2013).

Tissue-specific expression profiling revealed a clear division of labor among *LbaSOD* members during fruit development. The early-peaking genes, *LbaSOD2* and *LbaSOD5*, likely support the intense metabolic activity associated with fruit set and initial cell division, whereas the progressively upregulated *LbaSOD6*, *LbaSOD9*, and *LbaSOD10* may protect against oxidative stress linked to ripening-related metabolic shifts and pigment accumulation. This temporal partitioning is particularly relevant for wolfberry, where the fruit represents the primary organ of economic value; ROS management during fruit development directly influences quality traits and antioxidant content (Jimenez et al., 2002; Mellidou and Kanellis, 2017). The distinct expression patterns also suggest that different SOD members may be regulated by distinct developmental programs, potentially involving fruit-specific transcription factors that remain to be identified.

Under abiotic stress conditions, *LbaSOD*s exhibited subfamily-specific response dynamics, indicating a compartmentalized stress defense strategy. The rapid induction of chloroplast-localized Fe-SODs (*LbaSOD9* and *LbaSOD10*) under salt stress aligns with the role of chloroplasts as a major site of ROS generation during ionic stress (Miller et al., 2010). Conversely, the pronounced suppression of these same Fe-SOD genes at 24 hours of drought stress suggests targeted downregulation, possibly to redirect metabolic resources toward osmolyte synthesis or to prevent excessive H_2_O_2_ accumulation when Calvin cycle activity is reduced (Suzuki et al., 2011). The more modest and fluctuating responses of Cu/Zn-SOD and Mn-SOD members under salt and drought stress may reflect their roles in maintaining basal ROS scavenging in the cytosol and mitochondria, rather than mediating acute stress responses.

Among the five phytohormones tested, the approximately 60-fold induction of *LbaSOD5* by MeJA (**Figure 12**) is consistent with the well-established role of jasmonate signaling in orchestrating antioxidant defense during wounding and pathogen attack (Devoto and Turner, 2003; Sasaki-Sekimoto et al., 2005). In grape, MeJA enhances the accumulation of pharmacologically active compounds, such as polysaccharides and carotenoids (Zhang et al., 2024), a function that may also be conserved in wolfberry. The concurrent upregulation of *LbaSOD5* likely mitigates the ROS burst associated with increased metabolic flux, thereby protecting cellular integrity during fruit maturation. This suggests a coordinated regulatory node in which *LbaSOD5* acts as a key mediator linking jasmonate signaling to both oxidative stress tolerance and the production of high-quality medicinal berry. The sustained high-level expression of *LbaSOD2*, *LbaSOD5*, and *LbaSOD6* throughout the MeJA treatment period indicates that these genes are integral components of a prolonged jasmonate-mediated defense program rather than transient responders. SA treatment also induced strong and sustained expression of several SOD members, particularly *LbaSOD5* (~18-fold) and *LbaSOD9/10* (>10-fold), consistent with the crosstalk between SA signaling and redox regulation during systemic acquired resistance (Jiang and Zhang, 2002; Miura and Tada, 2014). Notably, the initial suppression followed by activation observed for many genes under SA and IAA treatments suggests a biphasic transcriptional reprogramming mechanism that may enable the plant to temporarily prioritize one defense pathway before engaging in another.

The most striking finding of this study was the exceptionally strong and temporally dynamic ABA response of the *LbaSOD* family. The induction magnitudes observed for *LbaSOD5* (2.5 × 10^5^-fold) and *LbaSOD10* (6 × 10^5^-fold) at 6 hours are, to our knowledge, among the highest reported for SOD genes in any plant species under similar experimental conditions. Although ABA-induced SOD upregulation has been documented across diverse species, including rice and *Arabidopsis* (Tsugane et al., 1999; Wang et al., 2005), the reported fold changes typically range from 2- to 20-fold. The exceptional magnitude observed here may reflect a highly amplified ABA–ROS regulatory response in wolfberry, potentially associated with its adaptation to the arid and semi-arid environments of northwestern China, where ABA-mediated drought tolerance is likely under strong selective pressure. The biphasic expression pattern exhibited by *LbaSOD4*, *LbaSOD5*, and *LbaSOD6*, with a primary peak at 6 hours, a subsequent decline, and a secondary increase at 24 hours, further suggests the involvement of multiple temporally separated regulatory processes, potentially including an early, direct ABA-responsive element-mediated transcriptional activation phase and a later feedback-regulated or secondary signaling-mediated response (Fujita et al., 2011). The preferential induction of chloroplast-localized Fe-SODs (*LbaSOD9* and *LbaSOD10*) by ABA highlights the chloroplast as a key regulatory compartment for ABA-mediated ROS homeostasis, consistent with the established role of ABA in regulating stomatal closure and photosynthetic responses under water deficit.

Notably, not all *LbaSOD* members responded uniformly to ABA: *LbaSOD1* was consistently suppressed, declining to approximately 20% of baseline at 24 hours. This differential regulation within a single gene family under the same stimulus is particularly informative, arguing against a simple, uniform ABA-responsive transcriptional program and instead supporting a model in which ABA signaling selectively activates or represses individual SOD isoforms according to their subcellular localization and the specific ROS detoxification requirements imposed by stress conditions. The suppression of *LbaSOD1* (a Cu/Zn-SOD), together with the substantial induction of Fe-SODs, may indicate a stress-induced redistribution of antioxidant capacity from the cytosol toward chloroplasts during ABA signaling. This hypothesis requires further validation through biochemical characterization and subcellular localization analyses.

Two family members, *LbaSOD3* and *LbaSOD7*, were undetectable across all tested tissues, developmental stages, and treatment conditions. Although their CDSs and conserved domains appear intact based on *in silico* analyses, the absence of detectable expression suggests either transcriptional silencing resembling pseudogenization or activation only under highly specific conditions not examined in this study, such as specific developmental stages, biotic interactions, or combined stress scenarios. This finding highlights an important consideration in functional genomics: genome annotation and *in silico* prediction alone cannot establish biological activity, and comprehensive experimental validation across diverse environmental and developmental contexts is required to distinguish functional genes from genomically intact but transcriptionally inactive loci.

This study had several limitations. First, our expression analysis was conducted at the transcript level using qRT-PCR; however, protein abundance and enzymatic activity were not measured, and post-translational regulation, which is known to modulate SOD activity independently of transcript levels, was not assessed. Second, the extraordinarily high fold-change values reported for *LbaSOD5* and *LbaSOD10* under ABA treatment, while biologically significant, should be interpreted with caution because the 2^ΔΔCt^ method can result in inflated relative expression estimates when baseline expression is very low. Absolute transcript abundance measurements would provide a complementary and more quantitative evaluation. Third, our hormone treatments used a single concentration (100 μM) applied to whole seedlings; future dose-response experiments and tissue-specific analyses will further refine our understanding of the regulatory dynamics. Finally, the functional significance of the observed expression patterns remains largely correlative, and direct functional validation through overexpression, silencing, or CRISPR-based mutagenesis in either wolfberry or heterologous systems is necessary to establish causal relationships between specific *LbaSOD* members and stress tolerance phenotypes.

Looking forward, several research directions emerge from this work. The exceptionally strong ABA–SOD regulatory axis in wolfberry warrants further mechanistic investigation. Chromatin immunoprecipitation assays can identify the transcription factors that bind to the ABRE motifs in the promoters of *LbaSOD5* and *LbaSOD10*, whereas promoter deletion and mutagenesis experiments can define the specific *cis*-regulatory regions responsible for the observed induction levels. The biphasic ABA response also calls for integrated time-resolved transcriptomic and proteomic analyses to identify the secondary signals driving the expression rebound at 24 hours. From an applied perspective, *LbaSOD* members exhibiting strong and specific stress or hormone responsiveness, particularly *LbaSOD5* (MeJA, ABA, and SA), *LbaSOD10* (ABA, salt, and MeJA), and *LbaSOD6* (ABA and cold), represent promising candidates for genetic improvement of stress tolerance in wolfberry and potentially other *Solanaceae* crops through marker-assisted selection or transgenic approaches. Comparative functional studies across *Solanaceae* would further clarify whether the exceptional ABA responsiveness observed here is unique to wolfberry or represents a more broadly shared characteristic of the family.

## 5 Conclusion

This study presents the first comprehensive genome-wide characterization of the SOD gene family in wolfberry, identifying 10 *LbaSOD* genes classified into three subfamilies with conserved structures and motifs. Segmental duplication was the primary driver of gene family expansion, and promoter analysis revealed numerous stress- and hormone-responsive *cis*-elements. Expression profiling across tissues, developmental stages, abiotic stresses, and five phytohormones revealed a highly diversified regulatory architecture, in which individual *LbaSOD* members are deployed in a stimulus-, subfamily-, and temporal-specific manner. A key finding was the exceptionally strong ABA-mediated induction of *LbaSOD5*, *LbaSOD10*, and *LbaSOD6*, together with the selective suppression of *LbaSOD1*, demonstrating that ABA exerts differential regulatory effects on distinct SOD isoforms within a single family. Furthermore, the transcriptional silencing of *LbaSOD3* and *LbaSOD7* under all conditions further suggests potential regulatory silencing rather than confirmed pseudogenization. Beyond providing a foundational resource for the wolfberry research community, this study identified specific *LbaSOD* candidates for functional validation and highlighted the ABA–SOD regulatory axis as a priority target for future mechanistic studies. Elucidating the molecular basis of the exceptional ABA responsiveness of wolfberry SODs, characterizing their post-translational regulation, and translating these insights into genetic improvement strategies for stress tolerance represent important directions for future investigations. Future studies should prioritize (1) subcellular localization of key isoforms, (2) functional validation through overexpression and gene-silencing approaches in wolfberry or suitable heterologous systems, (3) enzymatic activity assays and ROS quantification, and (4) mechanistic dissection of the ABA-responsive regulatory network using yeast one-hybrid and dual-luciferase assays.

## Data Availability

The wolfberry genome datasets used inthe current study are available in the NCBI database (https://www.ncbi.nlm.nih.gov/bioproject/PRJNA640228).The RNA-seq data used in this study were downloaded from the NCBI Database (PRJNA845109). The qPCR data can be found in the **Supplementary Material**.

## References

[B1] Amagase H. Farnsworth N. R. (2011). A review of botanical characteristics, phytochemistry, clinical relevance in efficacy and safety of *Lycium barbarum*fruit (Goji). Food Res. Int. 44, 1702–1717. doi: 10.1016/j.foodres.2011.03.027 42574925

[B2] Apel K. Hirt H. (2004). Reactive oxygen species: metabolism, oxidative stress, and signal transduction. Annu. Rev. Plant Biol. 55, 373–399. doi: 10.1146/annurev.arplant.55.031903.141701 15377225

[B3] Bucheli P. Vidal K. Shen L. Gu Z. Zhang C. Miller L. E. . (2011). Goji berry effects on macular characteristics and plasma antioxidant levels. Optometry Vision Sci. 88, 257–262. doi: 10.1097/OPX.0b013e318205a18f 21169874

[B4] Cannon S. B. Mitra A. Baumgarten A. Young N. D. May G. (2004). The roles of segmental and tandem gene duplication in the evolution of large gene families in *Arabidopsis thaliana*. BMC Plant Biol. 4, 10. doi: 10.1186/1471-2229-4-10 15171794 PMC446195

[B5] Cao Y. L. Li Y. L. Fan Y. F. Li Z. Yoshida K. Wang J. Y. . (2021). Wolfberry genomes and the evolution of *Lycium (Solanaceae)*. Commun. Biol. 4, 671. doi: 10.1038/s42003-021-02152-8 34083720 PMC8175696

[B6] Chahidi M. El Faqer A. Rabeh K. Belkadi B . (2025). Genome-wide survey of superoxide dismutase (SOD) genes in *Argania spinosa* L. an endemic tree species. Discov. Plants 2, 362. doi: 10.1007/s44372-025-00379-x 30311153

[B7] Chen C. J. Chen H. Zhang Y. Thomas H. R. Frank M. H. He Y. H. . (2020). Tbtools: an integrative toolkit developed for interactive analyses of big biological data. Mol. Plant 13, 1194–1202. doi: 10.1016/j.molp.2020.06.009 32585190

[B8] Dat J. F. Lopez-Delgado H. Foyer C. H. Scott I. M. (1998). Parallel changes in H_2_O_2_ and catalase during thermotolerance induced by salicylic acid or heat acclimation in mustard seedlings. Plant Physiol. 116, 1351–1357. doi: 10.1104/pp.116.4.1351 9536052 PMC35042

[B9] Devoto A. Turner J. G. (2003). Regulation of jasmonate-mediated plant responses in *Arabidopsis*. Ann. Bot. 92, 329–337. doi: 10.1093/aob/mcg151 12871847 PMC4257513

[B10] Feng K. Wang R. (2019). “ Superoxide dismutases in plants: Role in abiotic stress tolerance and signaling,” in Reactive Oxygen, Nitrogen and Sulfur Species in Plants: Production, Metabolism, Signaling and Defense Mechanisms. Eds. Gill T. J. G. Miller M. M. ( John Wiley & Sons), 57–80.

[B11] Feng K. Yu J. Cheng Y. Ruan M. Wang R. Ye Q. . (2016). The SOD gene family in tomato: identification, phylogenetic relationships, and expression patterns. Front. Plant Sci. 7, 1279. doi: 10.3389/fpls.2016.01279 27625661 PMC5003820

[B12] Fujita Y. Fujita M. Shinozaki K. Yamaguchi-Shinozaki K. (2011). ABA-mediated transcriptional regulation in response to osmotic stress in plants. J. Plant Res. 124, 509–525. doi: 10.1007/s10265-011-0412-3 21416314

[B13] Gill S. S. Tuteja N. (2010). Reactive oxygen species and antioxidant machinery in abiotic stress tolerance in crop plants. Plant Physiol. Biochem. 48, 909–930. doi: 10.1016/j.plaphy.2010.08.016 20870416

[B14] Huo C. He L. Yu T. Ji X. Li R. Zhu S. . (2022). The superoxide dismutase gene family in *nicotiana tabacum*: genome-wide identification, characterization, expression profiling and functional analysis in response to heavy metal stress. Front. Plant Sci. 13, 904105. doi: 10.3389/fpls.2022.904105 35599861 PMC9121019

[B15] Hyun T. K. (2025). The superoxide dismutase family in balloon flower (*Platycodon grandiflorus*): phylogenetic relationships, structural characteristics, and expression patterns. Curr. Issues Mol. Biol. 47, 351. doi: 10.3390/cimb47050351 40699750 PMC12109774

[B16] Inbaraj B. S. Lu H. Hung C. F. Wu W. B. Lin C. L. Chen B. H. (2008). Determination of carotenoids and their esters in fruits of *Lycium barbarum* Linnaeus by HPLC–DAD–APCI–MS. J. Pharm. Biomed. Anal. 47, 812–818. doi: 10.1016/j.jpba.2008.04.001 18486400

[B17] Jiang M. Zhang J. (2002). Water stress‐induced abscisic acid accumulation triggers the increased generation of reactive oxygen species and up‐regulates the activities of antioxidant enzymes in maize leaves. J. Exp. Bot. 53, 2401–2410. doi: 10.1093/jxb/erf090 12432032

[B18] Jimenez A. Creissen G. Kular B. Firmin J. Robinson S. Verhoeyen M. . (2002). Changes in oxidative processes and components of the antioxidant system during tomato fruit ripening. Planta 214, 751–758. doi: 10.1007/s004250100667 11882944

[B19] Kliebenstein D. J. Monde R. A. Last R. L. (1998). Superoxide dismutase in *Arabidopsis*: an eclectic enzyme family with disparate regulation and protein localization. Plant Physiol. 118, 637–650. doi: 10.1104/pp.118.2.637 9765550 PMC34840

[B20] Li X. M. Ma Y. L. Liu X. J. (2007). Effect of the *Lycium barbarum* polysaccharides on age-related oxidative stress in aged mice. J. Ethnopharmacol. 111, 504–511. doi: 10.1016/j.jep.2006.12.024 17224253

[B21] Liang W. Zhang Z. Yao N. Wang B. Yu W. Zhu Q. . (2025). Glycolysis and signal transduction participate in *Lycium barbarum*in response to NaCl stress through protein phosphorylation. BMC Plant Biol. 25, 405. doi: 10.1186/s12870-025-06402-3 40165053 PMC11956257

[B22] Liu J. Xu L. Shang J. Hu X. Yu H. Wu H. . (2021). Genome-wide analysis of the maize superoxide dismutase (SOD) gene family reveals important roles in drought and salt responses. Genet. Mol. Biol. 44, e20210035. doi: 10.1590/1678-4685-GMB-2021-0035 34606562 PMC8493800

[B23] Livak K. J. Schmittgen T. D. (2001). Analysis of relative gene expression data using realtime quantitative PCR and the 2^(-ΔΔCT) Method. Methods 25, 402–408. doi: 10.1006/meth.2001.1262 11846609

[B24] Lu W. Duanmu H. Qiao Y. Jin X. Yu Y. Yu L. . (2020). Genome-wide identification and characterization of the soybean SOD family during alkaline stress. PeerJ 8, e8457. doi: 10.7717/peerj.8457 32071807 PMC7007734

[B25] Mishra P. Sharma P. (2019). Superoxide dismutases (SODs) and their role in regulating abiotic stress induced oxidative stress in plants. In: Hasanuzzaman M. Fotopoulos V. Nahar K. Fujita M. (eds), Reactive Oxygen, Nitrogen and Sulfur Species in Plants: Production, Metabolism, Signaling and Defense Mechanisms. Hoboken, NJ: John Wiley & Sons, 57–80. doi: 10.1002/9781119468677.ch3

[B26] Mellidou I. Kanellis A. K. (2017). Genetic control of ascorbic acid biosynthesis and recycling in horticultural crops. Front. Chem. 5, 50. doi: 10.3389/fchem.2017.00050 28744455 PMC5504230

[B27] Miller A. F. (2012). Superoxide dismutases: Ancient enzymes and new insights. FEBS Lett. 586, 585–595. doi: 10.1016/j.febslet.2011.10.048 22079668 PMC5443681

[B28] Miller G. Schlauch K. Tam R. Cortes D. Torres M. A. Shulaev V. . (2009). The plant NADPH oxidase RBOHD mediates rapid systemic signaling in response to diverse stimuli. Sci. Signaling 2, ra45. doi: 10.1126/scisignal.2000448 19690331

[B29] Miller G. Suzuki N. Ciftci-Yilmaz S. Mittler R. (2010). Reactive oxygen species homeostasis and signalling during drought and salinity stresses. Plant Cell Environ. 33, 453–467. doi: 10.1111/j.1365-3040.2009.02041.x 19712065

[B30] Mittler R. (2002). Oxidative stress, antioxidants and stress tolerance. Trends Plant Sci. 7, 405–410. doi: 10.1016/s1360-1385(02)02312-9 12234732

[B31] Mittler R. (2017). ROS are good. Trends Plant Sci. 22, 11–19. doi: 10.1016/j.tplants.2016.08.002 27666517

[B32] Mittler R. Vanderauwera S. Suzuki N. Miller G. Tognetti V. B. Vandepoele K. . (2011). ROS signaling: the new wave? Trends Plant Sci. 16, 300–309. doi: 10.1016/j.tplants.2011.03.007 21482172

[B33] Miura K. Tada Y. (2014). Regulation of water, salinity, and cold stress responses by salicylic acid. Front. Plant Sci. 5, 4. doi: 10.3389/fpls.2014.00004 24478784 PMC3899523

[B34] Nakashima K. Yamaguchi-Shinozaki K. (2013). ABA signaling in stress-response and seed dormancy. Plant Cell Rep. 32, 959–970. doi: 10.1007/s00299-013-1418-1 23535869

[B35] Orozco-Cárdenas M. L. Narváez-Vásquez J. Ryan C. A. (2001). Hydrogen peroxide acts as a second messenger for the induction of defense genes in tomato plants in response to wounding, systemin, and methyl jasmonate. Plant Cell 13 (1), 179–191. doi: 10.1105/tpc.13.1.179 11158538 PMC102208

[B36] Potterat O. (2010). Goji (*Lycium barbarum* and *L. chinense*): Phytochemistry, pharmacology and safety in the perspective of traditional uses and recent popularity. Planta Med. 76, 7–19. doi: 10.1055/s-0029-1186218 19844860

[B37] Rudić J. Dragićević M. B. Momčilović I. Simonović A. D. Pantelić D. (2022). In silico study of superoxide dismutase gene family in potato and effects of elevated temperature and salicylic acid on gene expression. Antioxidants 11, 488. doi: 10.3390/antiox11030488 35326138 PMC8944489

[B38] Sasaki-Sekimoto Y. Taki N. Obayashi T. Aono M. Matsumoto F. Sakurai N. . (2005). Coordinated activation of metabolic pathways for antioxidants and defence compounds by jasmonates and their roles in stress tolerance in *Arabidopsis*. Plant J. 44, 653–668. doi: 10.1111/j.1365-313X.2005.02560.x 16262714

[B39] Song Y. Xie X. Wang Y. Gao W. Huang H. Cao F. . (2024). Identification of superoxide dismutase (SOD) gene family in ginkgo (*Ginkgo biloba* L.) and role of *gbSOD8* in response to salt stress. Forests 15, 2141. doi: 10.3390/f15122141 30654563

[B40] Suzuki N. Miller G. Morales J. Shulaev V. Torres M. A. Mittler R. (2011). Respiratory burst oxidases: the engines of ROS signaling. Curr. Opin. Plant Biol. 14, 691–699. doi: 10.1016/j.pbi.2011.07.014 21862390

[B41] Tang H. Bowers J. E. Wang X. Ming R. Alam M. Paterson A. H. (2008). Synteny and collinearity in plant genomes. Science 320, 486–488. doi: 10.1126/science.1153917 18436778

[B42] The Tomato Genome Consortium (2012). The tomato genome sequence provides insights into fleshy fruit evolution. Nature 485, 635–641. doi: 10.1038/nature11119 22660326 PMC3378239

[B43] Tsugane K. Kobayashi K. Niwa Y. Ohba Y. Wada K. Kobayashi H. (1999). A recessive *Arabidopsis* mutant that grows photoautotrophically under salt stress shows enhanced active oxygen detoxification. Plant Cell 11, 1195–1206. doi: 10.1105/tpc.11.7.1195 10402422 PMC144266

[B44] Tyagi S. Sharma S. Taneja M. Shumayla Kumar R. Sembi J. K. . (2017). Superoxide dismutases in bread wheat (*Triticum aestivum* L.): Comprehensive characterization and expression analysis during development and, biotic and abiotic stresses. Agri Gene 6, 1–13. doi: 10.1016/j.aggene.2017.08.003 42574925

[B45] Verma D. Lakhanpal N. Singh K. (2019). Genome-wide identification and characterization of abiotic-stress responsive *SOD* (*superoxide dismutase*) gene family in *Brassica juncea* and *B. rapa*. BMC Genomics 20, 227. doi: 10.1186/s12864-019-5593-5 30890148 PMC6425617

[B46] Wang F. Z. Wang Q. B. Kwon S. Y. Kwak S. S. Su W. A. (2005). Enhanced drought tolerance of transgenic rice plants expressing a pea manganese superoxide dismutase. J. Plant Physiol. 162, 465–472. doi: 10.1016/j.jplph.2004.09.009 15900889

[B47] Wu H. T. He X. J. Hong Y. K. Ma T. Xu Y. P. Li H. H. (2010). Chemical characterization of *lycium barbarum* polysaccharides and its inhibition against liver oxidative injury of high-fat mice. Int. J. Biol. Macromol 46, 540–543. doi: 10.1016/j.ijbiomac.2010.02.010 20193709

[B48] Xia X. J. Zhou Y. H. Shi K. Zhou J. Foyer C. H. Yu J. Q. (2015). Interplay between reactive oxygen species and hormones in the control of plant development and stress tolerance. J. Exp. Bot. 66, 2839–2856. doi: 10.1093/jxb/erv089 25788732

[B49] Xu X. Wang Q. Li W. Q. Hu T. X. Wang Q. Q. Yin Y. . (2022). Overexpression of *SlBBX17* affects plant growth and enhances heat tolerance in tomato. Int. J. Biol. Macromol 206, 799–811. doi: 10.1016/j.ijbiomac.2022.03.080 35307463

[B50] Yadav S. Gill S. S. Passricha N. Gill R. Badhwar P. Anjum N. A. . (2019). Genome-wide analysis and transcriptional expression pattern-assessment of superoxide dismutase (SOD) in rice and *Arabidopsis* under abiotic stresses. Plant Gene 17, 100165. doi: 10.1016/j.plgene.2018.10.001 42574925

[B51] Yamaguchi-Shinozaki K. Shinozaki K. (2005). Organization of cis-acting regulatory elements in osmotic- and cold-stress-responsive promoters. Trends Plant Sci. 10, 88–94. doi: 10.1016/j.tplants.2004.12.012 15708346

[B52] Yao R. Heinrich M. Weckerle C. S. (2018). The genus *Lycium* in food and medicine: A botanical, ethnobotanical, and historical review. J. Ethnopharmacol. 212, 50–66. doi: 10.1016/j.jep.2017.10.010 29042287

[B53] Yu S. Wang C. Wang Q. Sun Q. Zhang Y. Dong J. . (2023). Identification and analysis of SOD family genes in peanut (*Arachis hypogaea* L.) and their potential roles in stress responses. Agronomy 13, 1959. doi: 10.3390/agronomy13081959 30654563

[B54] Zhang K. K. Zhang J. X. Zheng T. Y. Gu W. J. Zhang Y. Y. Li W. P. . (2024). Preharvest application of MeJA enhancing the quality of postharvest grape berries via regulating terpenes biosynthesis and phenylpropanoid metabolisms. Food Chem. 438, 0308–8146. doi: 10.1016/j.foodchem.2023.137958 38000159

[B55] Zhao J. H. Li H. X. Zhang C. Z. An W. Yin Y. Wang Y. J. . (2018). Physiological response of four wolfberry (*Lycium* Linn.) species under drought stress. J. Integr. Agric. 17, 603–612. doi: 10.1016/S2095-3119(17)61754-4

[B56] Zhou Y. Li S. Ran S. Xu Y. Hou M. Han M. . (2024). Genome-wide identification and characterization of the superoxide dismutase (*SOD*) gene family in Pakchoi and the role of the *BchFSD2* gene in the salt stress toleran. Agronomy 14, 384. doi: 10.3390/agronomy14020384 30654563

