# Supplementary material for: Genome-wide identification of the superoxide dismutase gene family in Lycium barbarum and their expression profiles under abiotic stress and phytohormone treatment
Source: Front Plant Sci. 2026 Aug 27;17:1897912. doi: 10.3389/fpls.2026.1897912 (PMC13560488; doi:10.3389/fpls.2026.1897912)
Supplement: Supplementary file 1 [file Supplementaryfile1.docx]

**Supplementary Figure 1.** Chromosomal localization of *LbaSOD* genes. The figure shows the distribution of *LbaSOD* genes from *L. barbarum* across 10 chromosomes. The scale on the left indicates the chromosome length. The scale is in Megabase (Mb).

(a)

(b)

**Supplementary Figure 2..** Synteny analysis of wolfberry SOD genes. (a) Chromosomal localization and synteny of SOD genes in wolfberry. SOD genes were mapped to distinct chromosomes, with syntenic gene pairs linked by red lines. (b) Cross-species synteny analysis of SOD genes between wolfberry and tomato, potato, and Arabidopsis (red lines denote syntenic LbaSOD pairs).
